# TMEM160 inhibits KEAP1 to suppress ferroptosis and induce chemoresistance in gastric cancer

**DOI:** 10.1038/s41419-025-07621-0

**Published:** 2025-04-13

**Authors:** Chunye Huang, Qinru Zeng, Jingyi Chen, Qin Wen, Weilun Jin, Xiaofeng Dai, Ruiwen Ruan, Hongguang Zhong, Yang Xia, Zhipeng Wu, Ruixuan Huang, Jianxi Zhang, Yangyang Yao, Li Li, Wan Lei, Jianping Xiong, Jun Deng

**Affiliations:** 1 Department of Oncology, The First Affiliated Hospital, Jiangxi Medical College, Nanchang University, Nanchang, China; 2 Jiangxi Key Laboratory for Individual Cancer Therapy, Nanchang, China; 3 Postdoctoral Innovation Practice Base, The First Affiliated Hospital of Nanchang University, Nanchang, China

**Keywords:** Gastrointestinal cancer, Cell death

## Abstract

Chemoresistance is the most significant challenge affecting the clinical efficacy of the treatment of patients with gastric cancer (GC). Here we reported that transmembrane protein 160 (TMEM160) suppressed ferroptosis and induced chemoresistance in GC cells. Mechanistically, TMEM160 recruited the E3 ligase TRIM37 to promote K48-linked ubiquitination and degradation of KEAP1, thereby activating NRF2 and transcriptionally upregulating the target genes *GPX4* and *SLC7A11* to inhibit ferroptosis. Further in vitro and in vivo experiments demonstrated that the combination of TMEM160 targeting and chemotherapy had a synergistic inhibitory effect on the growth of GC cells, which was partially NRF2-dependent. Moreover, TMEM160 and NRF2 protein levels were markedly overexpressed in GC tissues, and their co-overexpression was an independent factor for poor prognosis. Collectively, these findings indicate that TMEM160, as a pivotal negative regulator of ferroptosis, exerts a crucial influence on the chemoresistance of GC through the TRIM37-KEAP1/NRF2 axis, providing a potential new prognostic factor and combination therapy strategy for patients with GC.

## Introduction

Gastric cancer (GC) stands as one of the most prevalent malignant tumors worldwide, ranking third in terms of cancer-related mortality, posing a severe threat to human health [1, 2]. The early diagnosis rate of GC in China is notably low, attributed to the lack of a comprehensive early screening program, and the five-year survival rate is less than 30%, indicating an abysmal prognosis for patients [3]. Owing to primary or acquired resistance to chemotherapy, up to 40–60% of patients experience recurrence or metastasis, rendering chemoresistance a significant clinical challenge that needs urgent resolution [4, 5]. Therefore, an in-depth exploration of the molecular mechanisms underlying chemoresistance in GC and the accurate prediction of chemotherapy efficacy are critical for guiding individualized clinical treatment and improving prognosis.

Ferroptosis is a novel form of regulated cell death, which is associated with various pathological conditions [6–9]. Several critical factors, such as glutathione peroxidase 4 (GPX4) and solute carrier family 7 member 11 (SLC7A11), play essential roles in modulating ferroptosis [10–12]. The Kelch-like ECH-associated protein 1 (KEAP1)/nuclear factor erythroid 2-related factor 2(NRF2) signaling pathway is a key regulator of the antioxidant response [13]. KEAP1 functions as a substrate adapter that recruits NRF2 into the Cul3-dependent E3 ubiquitin ligase complex, leading to the proteasomal degradation of NRF2 [13, 14]. NRF2 serves as a transcription factor that binds to the antioxidant response elements of *SLC7A11* and *GPX4* to suppress ferroptosis in cancer cells [15–20]. The KEAP1/NRF2 signaling pathway is abnormally activated in GC tissues [21, 22], however, the alteration rates of *KEAP1* or *NRF2* genes are very low in patients with GC [23]. Therefore, elucidating the precise regulatory mechanisms controlling the KEAP1-NRF2 pathway could provide novel insights into cancer development and potentially serve as an innovative therapeutic strategy for GC.

Transmembrane protein 160 (TMEM160) is a member of the TMEM family. Our previous studies found that TMEM160 is associated with poor prognosis in patients with colorectal cancer, as it can inhibit the ubiquitination and degradation of programmed death-ligand 1 (PD-L1) by competing with Speckle-type POZ protein (SPOP) to promote immune evasion and radioresistance [24]. Additionally, TMEM160 knockout leads to increased reactive oxygen species (ROS) production and induces a mitochondrial unfolded protein response, indicating that TMEM160 inhibits ROS production in cancer cells [25]. However, whether TMEM160 is involved in regulating ferroptosis in GC remains unclear.

In this study, we aimed to investigate the role of TMEM160 in the inhibition of ferroptosis and the promotion of malignant progression and chemoresistance in GC. The objective was to examine the underlying mechanism by examining the effect of TMEM160 on KEAP1. Additionally, we aimed to examine the potential of TMEM160 as a novel prognostic factor and combination therapy strategy for patients with GC.

## Materials and methods

### Patient samples and clinical data collection

Paraffin-embedded tissue samples were collected from 180 patients with GC who underwent surgical treatment without any systemic or local therapy from January 2017 to April 2019 and 89 patients with advanced GC who underwent first-line chemotherapy combined with immunotherapy from January 2020 to July 2024 at the First Affiliated Hospital of Nanchang University. All patients were diagnosed with primary GC and provided informed consent to participate in this study, which was approved by the Ethics Committee of The First Affiliated Hospital of Nanchang University (approval number: (2023)CDYFYYLK(02-068)) and was performed following the guidelines of the Declaration of Helsinki. The clinical information of the patients and the statistical information are summarized in Supplementary Tables 9 and 11. The detailed information of the patients used in our experiments is presented in Supplements 1 and 2.

### Cell culture

Human GC cell lines BGC-823 and HGC-27 were acquired from the Shanghai Institute of Cell Biology, China Academy of Sciences, and cultured in RPMI 1640 medium containing 10% fetal bovine serum (FBS). The SNU-216 cell line was purchased from Procell Life Science and Technology (Wuhan, China) and cultured similarly. HEK-293T cells were cultured in Dulbecco’s modified Eagle medium containing 10% FBS. All cells were maintained in a humidified incubator at 37°C with 5% CO_2_.

### RNA interference, lentivirus plasmid construction, and transfection

The cells were transfected with siRNAs and DNA plasmids using the TurboFect Transfection Reagent (R0531, Thermo Fisher Scientific Corp, Waltham, USA). The siRNAs were constructed by GenePharma(Shanghai, China). The plasmids, including HA-KEAP1, Flag-KEAP1, Myc-TMEM160, Flag-TRIM37, His-Ub (K48, K63), and His-Ub (WT) were obtained from GeneChem (Shanghai, China) and Miaoling Biological (Wuhan, China). TMEM160 shRNA lentivirus was designed and purchased from GeneChem. The cells were transfected with siRNAs and plasmids when the cell confluence reached 40–50% and 70–80%, respectively. Detailed sequences of all siRNAs, shRNAs, and lentiviruses are provided in Supplementary Table 1.

### RNA extraction and quantitative reverse transcription polymerase chain reaction (RT-qPCR) assay

RNA was extracted from cells using an RNA extraction reagent(R701-01, Vazyme Corp, Nanjing, China). Extracted RNA was reverse-transcribed into complementary DNA (cDNA)(AT341-01, TransGen Biotech Corp, Beijing, China). RT-qPCR was performed using a StepOnePlus Real-Time PCR System(11736059, Thermo Fisher Scientific Corp, Waltham, MA, USA). Each sample was tested in triplicate, and the PCR results were normalized to those of the control group using GAPDH. All experiments were repeated at least three times under identical conditions. The primer sequences are listed in Supplementary Table 2.

### Western blotting (WB)

Total proteins were extracted from the cells 48 h after transfection using RIPA buffer containing protease and phosphatase inhibitors at 4°C and were separated by sodium dodecyl sulfate polyacrylamide gel electrophoresis and transferred onto nitrocellulose membranes(PALL cat. no. 66485). The detailed experimental procedures have been described previously [26]. All experiments were repeated at least thrice under identical conditions. Information on the primary antibodies used in the WB is provided in Supplementary Table 3.

### Immunofluorescence (IF)

GC cells were subjected to IF analysis 48 h after transfection as previously described [24]. The colocalization of TMEM160, KEAP1, and TRIM37 in the cells was observed using a laser confocal microscope(Stellaris 5, Carl Zeiss, Jena, Germany). All experiments were repeated at least three times under identical conditions.

### Molecular docking

The 3D structure of KEAP1 was obtained from the Protein Data Bank (PDB). The 3D structural models of TMEM160 and TRIM37 were predicted using the UniProt database, and molecular docking analysis of the TMEM160, KEAP1, and TRIM37 structures was performed using the Discovery Studio software (BIOVIA Company, USA).

### Co-immunoprecipitation (Co-IP) assay

Exogenous Co-IP assays were performed on HEK-293T cells using a protein A/G Immunoprecipitation Kit (22202-100 Beaver Bio Corp, Suzhou, China) according to the manufacturer’s instructions. For endogenous Co-IP, GC cells lysates were incubated with magnetic beads conjugated to anti-TMEM160, anti-KEAP1, or anti-TRIM37 polyclonal antibodies at 4°C for 48 h. Proteins bound to the magnetic beads were analyzed by WB. The detailed experimental procedures were performed as previously described [27]. All experiments were repeated at least three times under identical conditions.

### Glutathione *S*-transferase (GST) pull-down assay

GST pull-down assays were performed using a GST Pull-Down Kit (M7006, Mabnus, Wuhan, China). Purified human proteins included TMEM160 (Ag24915, Proteintech, San Ying Biotechnology, China), GST-tagged protein (Ag0040, Proteintech), and KEAP1 fusion protein (Ag32533, Proteintech). The samples were analyzed by gel electrophoresis and WB. The experimental procedures were performed as previously described [27]. All experiments were repeated at least three times under identical conditions.

### Ubiquitination assay

BGC-823 and HEK-293T cells were transfected with HA-KEAP1, Myc-TMEM160, Flag-TRIM37, His-Ub (WT), His-Ub (K48), or His-Ub (K63) plasmids according to the experimental groups. Cells were treated with 20 μM MG132(HY-13259, MedChemExpress, USA) for 6 h before protein extraction, and proteins were collected 48 h after transfection. Cell lysates were incubated with magnetic beads conjugated to anti-HA, anti-Myc, or anti-Flag antibodies at 4°C for 48 h. Proteins bound to the magnetic beads were analyzed by WB. All experiments were repeated at least three times under identical conditions.

### Cycloheximide (CHX) half-life, MG132, and chloroquine (CQ) treatment assay

After 36 h of transfection, cells were treated with 100 μg/mL of CHX(HY-12320, MedChemExpress) for 0 h, 6 h, 12 h, 24 h, and 36 h. For the proteasome inhibitor MG132 and lysosome inhibitor CQ(HY-17589A, MedChemExpress) recovery experiments, cells were treated with 20 μM MG132 and 50 μM CQ for 6 and 12 h, respectively. The protein lysates were collected for WB analysis. All experiments were repeated at least three times under identical conditions.

### Lipid peroxidation assay

After 48 h of transfection, cells were treated with or without erastin for 24 h and then incubated with 5 μM BODIPY™ 581/591 C11(D3861, Invitrogen) at 37°C in the dark for 30 min. Then the samples were analyzed using a Mindray flow cytometer(BriCyte E6, Mindray, Shenzhen, China), and the data were analyzed using the FlowJo software (Becton Dickinson and Company, New Jersey, USA). All experiments were repeated at least three times under identical conditions.

### Malondialdehyde (MDA) assay

MDA levels were measured using the Lipid Peroxidation MDA Assay Kit(S0131S, Beyotime) according to the manufacturer’s instructions. After 48 h of transfection, the cells were treated with erastin for 24 h. The cell lysates were collected, and the MDA assay working solution was added. The solution was mixed and incubated at 100°C for 15 min. Then, the absorbance of each sample was measured with a microplate reader at 532 nm. The MDA concentrations were calculated based on the standard solutions with gradient MDA concentrations, and normalized to the protein quality. All experiments were repeated at least thrice under identical conditions.

### Intracellular Fe^2+^ assay

Intracellular iron levels were measured using the Cell Ferrous Iron Colorimetric Assay Kit(E-BC-K881-M, Elabscience Biotechnology) according to the manufacturer’s instructions. After 48 h of transfection, the cells were treated with erastin for 24 h. The cell lysates were collected, and chromogenic solution was added. The solution was mixed and incubated at 37°C for 10 min. Then, the absorbance of each sample was measured with a microplate reader at 593 nm. The iron concentrations were calculated based on the standard solutions with gradient iron concentrations, and normalized to the protein quality. All experiments were repeated at least three times under identical conditions.

### Glutathione (GSH) assay

GSH levels were measured using the GSH and GSSG Assay Kit (S0053, Beyotime) following the manufacturer’s instructions. After 48 h of transfection, cells were treated with erastin for 24 h. Cells were then collected, homogenized with protein removal reagent M, and incubated at 4°C for 5 min. After centrifugation, the supernatant was collected for total GSH measurement. A portion of the supernatant was mixed with GSH scavenging reagent and incubated at 25°C for 60 min to measure GSSG. Total GSH detection solution was added, incubated at room temperature for 25 min, and absorbance was measured at 412 nm. Total GSH and GSSG concentrations were calculated based on a gradient of GSSG standard solutions using the formula: GSH = Total GSH − GSSG × 2. Results were normalized to protein quality. All experiments were repeated at least three times under identical conditions.

### Transmission electron microscopy (TEM)

After 36 h of transfection, the cells were treated with erastin for 24 h. The cells were collected and fixed with an electron microscopy fixative. After washing with PBS, the cells were pre-embedded in a 1% agarose solution and fixed in 1% osmium tetroxide. After dehydration through a gradient of ethanol concentrations, the cells were infiltrated, embedded, and cut into 60–80 nm ultrathin sections. The sections were sequentially stained with 2% uranyl acetate and 2.6% lead citrate. Images were captured and analyzed using TEM.

### Apoptosis assay

After 48 h of transfection, cells were harvested using EDTA-free trypsin. The collected cells were incubated in 100 μL binding buffer with 5 μL Annexin V-FITC and 5 μL propidium iodide staining solution (A211-01, Vazyme, Nanjing, China) at room temperature in the dark for 10 min. Then the apoptosis rate was analyzed using a Mindray flow cytometer, and the data were processed using FlowJo software. All experiments were repeated at least three times under identical conditions.

### Cell proliferation, colony formation, and drug sensitivity assays

After transfection, cells were seeded in 96-well plates and treated with gradient concentrations of 5-fluorouracil (5-fu) (CAS No: 51-21-8, TargetMol), oxaliplatin(CAS No: 61825-94-3, Psaitong), or erastin(HY-15763, MedChemExpress). After 1–5 days of culturing, cells were incubated with Cell Counting Kit-8 (CCK-8)(GK10001, GlpBio) reagent for 2 h, and the absorbance at 450 nm was measured for cell proliferation assay. For drug sensitivity evaluation, absorbance readings via CCK-8 assay were obtained 24 h post-erastin treatment and 48 h after chemotherapy drug exposure. For colony formation assays, transfected cells were seeded into 6-well plates and cultured for 10–15 days. Colonies were then fixed with 4% paraformaldehyde (P0099, Beyotime), stained with crystal violet (C0121, Beyotime), and quantified using ImageJ software. All experiments were repeated at least three times under identical conditions.

### Migration, invasion, and wound-healing assays

After transfection, the cells were seeded into Transwell chambers for migration and invasion assays. For invasion assays, Transwell chambers were coated with Matrigel prior to cell seeding. After 48–72 h, the cells were fixed with 4% paraformaldehyde and stained with crystal violet. Wound healing assays were performed as previously described [28]. All experiments were repeated at least thrice under identical conditions.

### Immunohistochemistry (IHC) assays

IHC analysis was performed on paraffin-embedded tissue sections obtained from the 269 GC samples. The IHC results were evaluated by two pathologists. The IHC procedures were performed as previously described [24]. TMEM160 and NRF2 expression was quantified via IHC using a semi-quantitative scoring system integrating staining intensity (0: negative; 1: weak; 2: moderate; 3: strong) and the proportion of positive stained cells (1: ≤25%; 2: 26–50%; 3: 51–75%; 4: >75%). The composite IHC score was derived by multiplying these two parameters, with scores ≤6 classified as low expression and >6 as high expression.

### Establishment of GC CDX models and chemotherapy treatment

LV-shScr and LV-shTMEM160-#2 lentiviruses were used to establish CDX models with BGC-823 and HGC-27 cells. Four-week-old female BALB/c nude mice were randomly divided into four groups (*n* = 6 per group), and the cells were subcutaneously injected into the axilla. Once the subcutaneous tumors reached a size of 80 mm^3^, the mice were administered intraperitoneal injections of PBS or 5-fu every three days with a total of five injections (drug concentration: 40 mg/kg). After treatment, the mice were subjected to in vivo imaging. Tumor size was measured and recorded every three days. The tumor volume was calculated in mm^3^ using the following formula: *V* = *a* × *b*^2^/2, where *a* and *b* represent the length and width of the tumor, respectively. All animal experiments were approved by the Animal Ethics Committee of the First Affiliated Hospital of Nanchang University (approval number: CDYFY-IACUC-202208QR029).

### Establishment of patient-derived xenograft (PDX) in mice and lentivirus treatment

Fresh GC tumor tissues (F0 tumors) were obtained through surgery and subcutaneously injected into BALB/c nude mice. When PDX tumors grew to 800 mm^3^ (P0), the tumors were excised, cut into 3 × 3 × 3 mm pieces, and transplanted into the axilla of 4-week-old female BALB/c nude mice. When the tumors grew to approximately 150 mm^3^, the mice were randomly divided into control and experimental groups (*n* = 7 per group). LV-shScr and LV-shTMEM160-#2 lentiviruses were injected into the tumors of each group. The experimental procedures were the same as those described previously [27].

### Statistical analysis

Statistical analysis was performed using the SPSS software (version 26.0) and GraphPad Prism8. For comparisons between the two groups, continuous variables were analyzed using Student’s *t*-test, and categorical variables were analyzed using the chi-square test. A two-way analysis of variance (ANOVA) was used to compare multiple groups. Kaplan–Meier survival curves were plotted, and log-rank tests were used to analyze the data. *p* < 0.05 was considered statistically significant. All analyses were based on the data obtained from at least three independent experiments, and the data are presented as the means ± standard deviations (SDs).

## Results

### Systematic identification of TMEM160 as a key regulator of ferroptosis in GC cells

**Fig. 1:**
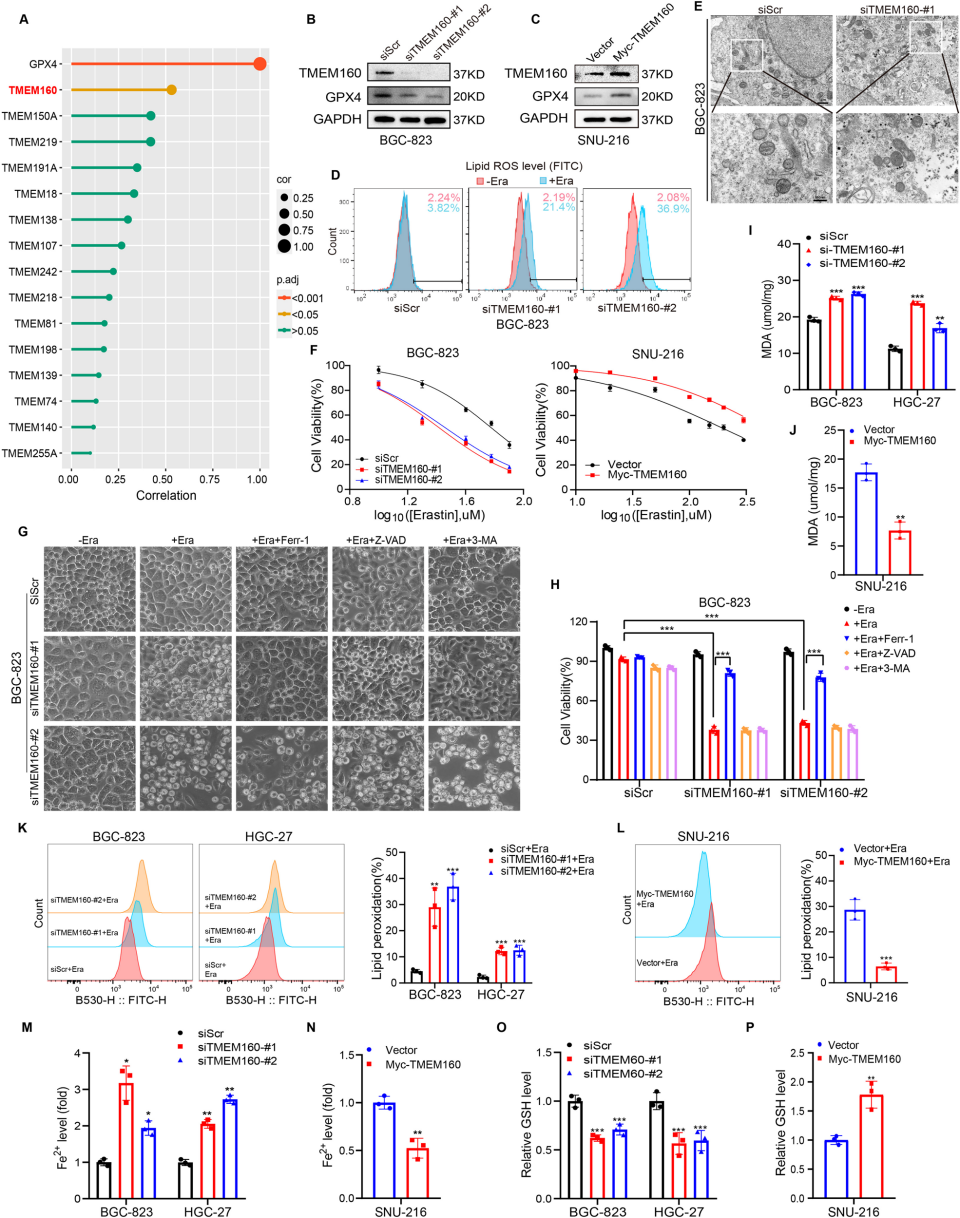
Systematic identification of TMEM160 as a key regulator of ferroptosis in GC. **A** Correlation analysis between GPX4 and TMEM family members in GC using the TCGA database; **B**, **C** Downregulation and upregulation of TMEM160 in BGC-823 and SNU-216 cells, respectively, detection of GPX4 expression by WB. **D** Lipid peroxidation measured by flow cytometry after C11-BODIPY staining in BGC-823 cells with TMEM160 depletion. **E** TEM analysis of BGC-823 cells with TMEM160 depletion treated with erastin, scale bar: 1 μm (top row) and 500 nm (bottom row). **F** Cell viability assessed by CCK-8 assay after treatment of TMEM160-depleted BGC-823 cells and TMEM160-overexpressing SNU-216 cells with different concentrations of erastin. **G** Representative light microscope images of TMEM160-depleted BGC-823 cells treated with erastin, erastin and Ferr-1, erastin and Z-VAD-FMK (Z-VAD), or erastin and 3-MA. **H** Bar graph showing the viability of TMEM160-depleted BGC-823 cells treated with erastin combined with Ferr-1, Z-VAD, or 3-MA. **I**, **J** MDA content detected after 24 h of erastin treatment in BGC-823 and HGC-27 cells with downregulation of TMEM160 and SNU-216 cells with upregulation of TMEM160. **K**, **L** Lipid peroxidation measured by flow cytometry after C11-BODIPY staining in BGC-823 and HGC-27 cells with downregulation of TMEM160, and SNU-216 cells with upregulation of TMEM160 after 24 h of erastin treatment. **M**, **N** Fe^2+^ content was detected after 24 h of erastin treatment in BGC-823 and HGC-27 cells, with downregulation of TMEM160 and SNU-216 cells with upregulation of TMEM160. **O**, **P** GSH content was detected after 24 h of erastin treatment in BGC-823 and HGC-27 cells, with downregulation of TMEM160 and SNU-216 cells with upregulation of TMEM160. Independent biological experiments were repeated at least three times, and the data are presented as the means ± SDs. Statistical differences are indicated by *p*-values of ^*^p < 0.05, ^**^p < 0.01, and ^***^p < 0.001.

Initially, we used the Cancer Genome Atlas (TCGA) database to analyze the expression of the ferroptosis-related genes *GPX4* and those belonging to the TMEM family in GC samples. The results showed that TMEM160 positively correlated with *GPX4*, exhibiting the strongest correlation (Fig. 1A and Supplementary Fig. 1A, B). Furthermore, Kyoto Encyclopedia of Genes and Genomes(KEGG) enrichment analysis using the LinkedOmics database indicated that TMEM160 was related to the GSH metabolism pathway (Supplementary Fig. 1C, D). These findings indicate that TMEM160 may be involved in regulating ferroptosis in GC.

To further elucidate the potential role of TMEM160 in the ferroptosis of GC cells, we found that knockdown of TMEM160 in BGC-823 cells reduced GPX4 protein levels, whereas overexpression of TMEM160 in SNU-216 cells increased GPX4 protein levels (Fig. 1B, C). Additionally, TMEM160 knockdown significantly upregulated erastin-induced lipid peroxidation in BGC-823 cells (Fig. 1D and Supplementary Fig. 1E), whereas it had no significant effect on apoptosis in GC cells (Supplementary Fig. 1F, G). TEM analysis further revealed that TMEM160 knockdown in GC cells resulted in mitochondrial shrinkage and increased membrane density(Fig. 1E), which are typical morphological features of ferroptosis [29, 30].

Functionally, TMEM160 overexpression inhibited erastin-induced death in SNU-216 cells, whereas its knockdown significantly promoted erastin-induced death in BGC-823 and HGC-27 cells (Fig. 1F, Supplementary Fig. 1H, and Supplementary Table 4). These effects were mitigated upon treatment with ferroptosis inhibitor Ferr-1 but not by the apoptosis inhibitor Z-VAD-FMK or the autophagy inhibitor 3-methyladenine (3-MA) (Fig. 1G, H), indicating that erastin-induced death in GC cells with TMEM160 knockdown was dependent on ferroptosis and not related to apoptosis or autophagy. The knockdown of TMEM160 increased MDA, lipid peroxidation, and Fe^2+^ levels and reduced GSH levels in GC cells(Fig. 1I, K, M, and O), whereas overexpression had the opposite effect (Fig. 1J, L, N, and P). In summary, these results suggested that TMEM160 is a key regulator of ferroptosis and can inhibit ferroptosis in GC cells.

### TMEM160 promotes malignant biological behaviors and chemoresistance in GC cells

**Fig. 2:**
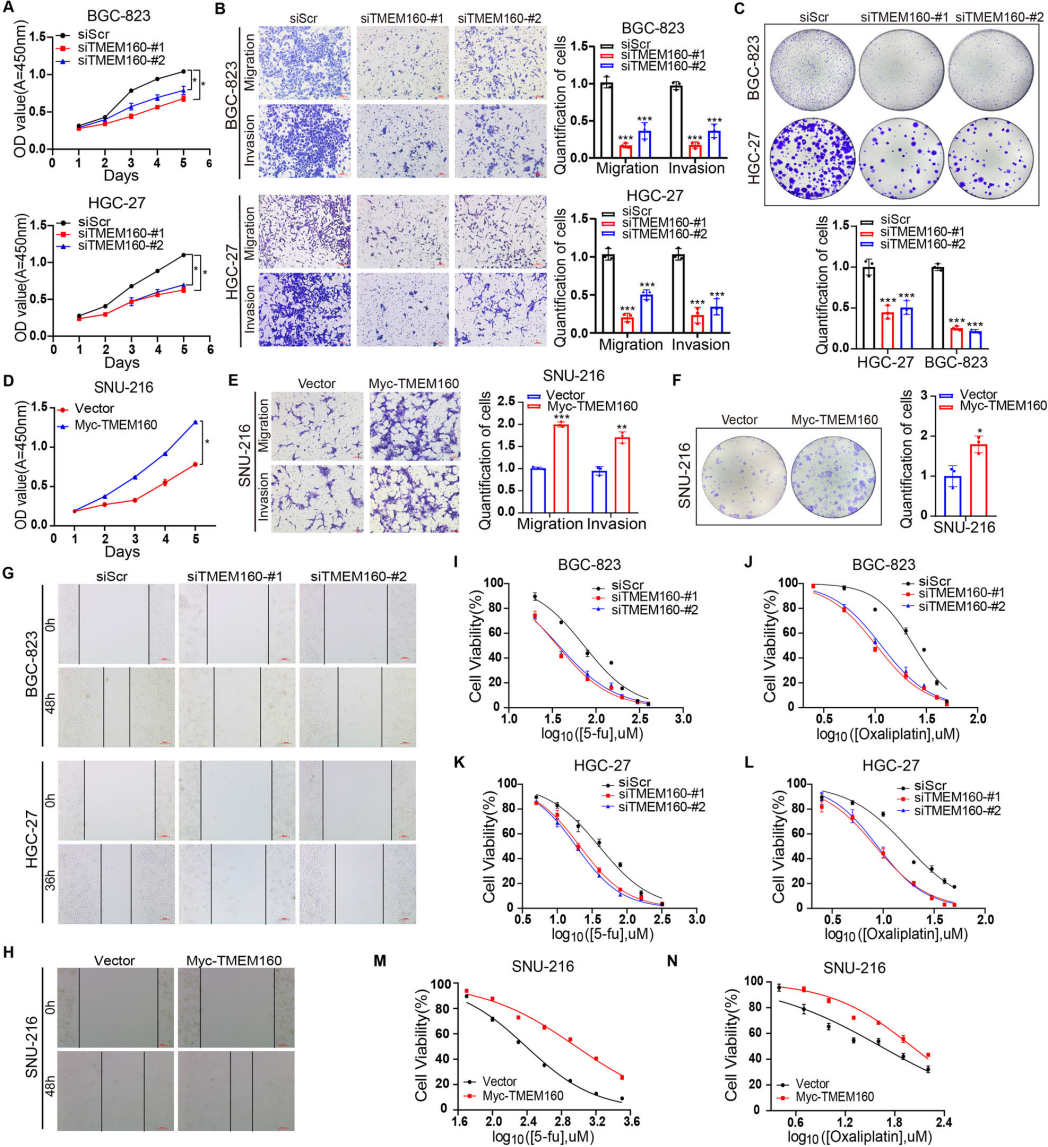
TMEM160 promoted malignant progression and chemoresistance in GC. **A** CCK-8 assays showing that downregulation of TMEM160 inhibited proliferation in BGC-823 and HGC-27 cells. **B**, **G** Transwell and wound healing assays showing that TMEM160 downregulation inhibited cell migration and invasion, scale bar: 100 μm. **C** Colony formation assay showing that TMEM160 downregulation inhibited colony formation ability. **D** CCK-8 assay showing that TMEM160 upregulation promoted cell proliferation in SNU-216 cells. **E**, **H** Transwell and wound healing assays showing that TMEM160 upregulation enhanced cell migration and invasion, scale bar: 100 μm. **F** Colony formation assay showing that TMEM160 upregulation enhanced colony formation ability. **I**–**L** Toxic proliferation assays showing that TMEM160 downregulation enhanced chemotherapy drug sensitivity. **M**, **N** Toxic proliferation assays showed that TMEM160 upregulation reduced chemotherapy drug sensitivity. Independent biological experiments were repeated at least three times, and the data are presented as the means ± SDs. Statistical differences are indicated by *p*-values of ^*^p < 0.05, ^**^p < 0.01, and ^***^p < 0.001.

To further understand the impact of TMEM160 on the biological behavior of GC cells in vitro, we stably overexpressed TMEM160 in SNU-216 cells and knocked down its expression in HGC-27 and BGC-823 cells; siTMEM160 (#1 and #2) and TMEM160 overexpression plasmids exhibited strong knockdown and overexpression efficiencies, respectively (Supplementary Fig. 2A–C). CCK8 and colony formation assays showed that TMEM160 knockdown inhibited the proliferation of HGC-27 and BGC-823 cells (Fig. 2A, C), whereas TMEM160 overexpression promoted the proliferation of SNU-216 cells (Fig. 2D, F). Wound healing, invasion, and migration assays indicated that TMEM160 knockdown inhibited the invasion and migration of HGC-27 and BGC-823 cells (Fig. 2B, G and Supplementary Fig. 2D, E), whereas TMEM160 overexpression promoted the invasion and migration of SNU-216 cells (Fig. 2E, H and Supplementary Fig. 2F). Cytotoxic proliferation assays revealed that TMEM160 knockdown increased the sensitivity of BGC-823 and HGC-27 cells to oxaliplatin and 5-fu (Fig. 2I–L and Supplementary Table 5), whereas TMEM160 overexpression reduced the sensitivity of SNU-216 cells to these chemotherapeutic drugs (Fig. 2M, N and Supplementary Table 5). In summary, these observations suggest that TMEM160 positively regulates the proliferation, invasion, migration, and chemoresistance in GC cells.

### Interaction between TMEM160 and the KELCH domain of KEAP1

**Fig. 3:**
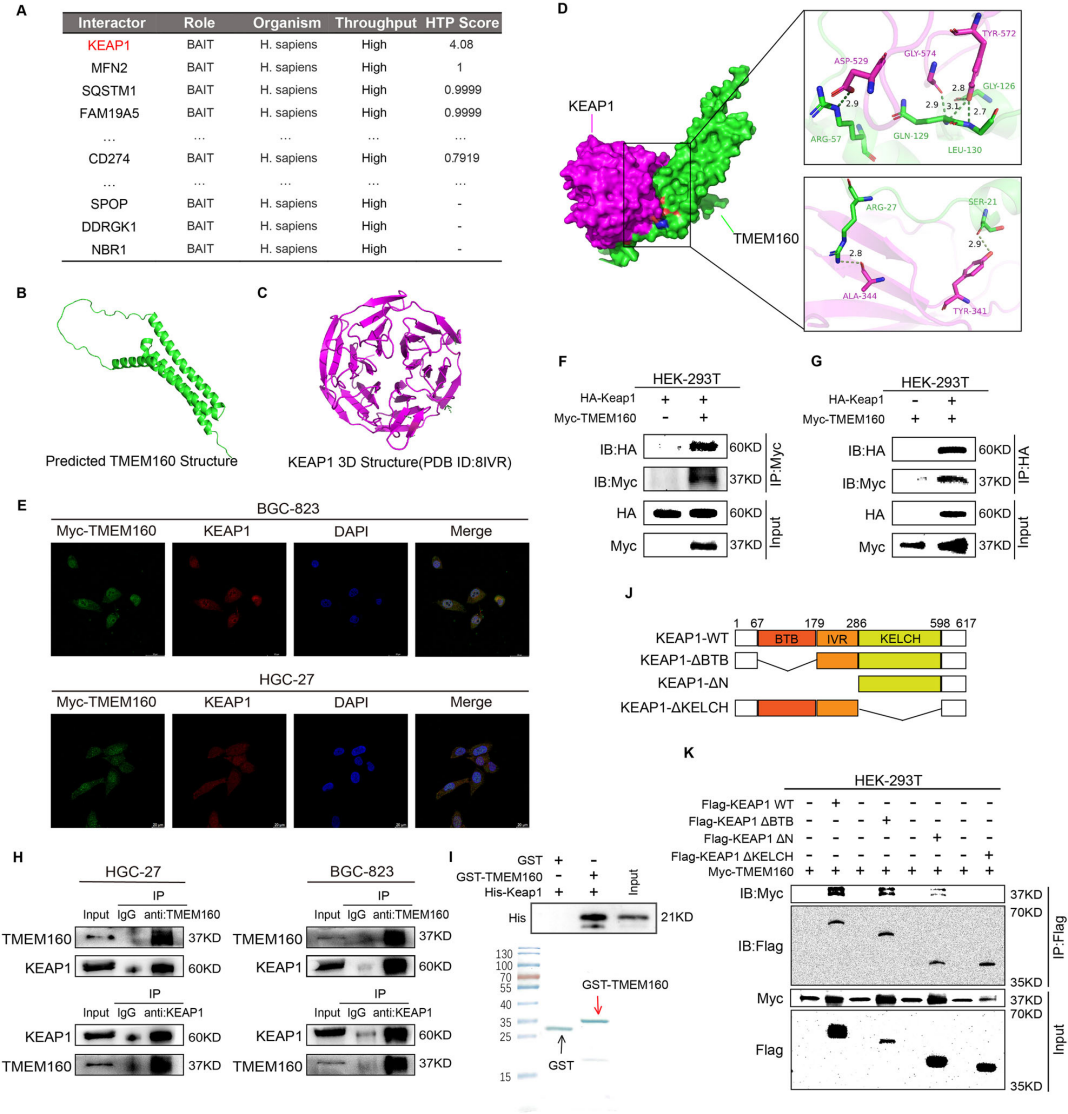
TMEM160 interacted with the KELCH domain of KEAP1. **A** Retrieval of TMEM160 interaction proteins using the BioGRID database. **B** Predicted 3D structural model of TMEM160 obtained from the UniProt database. **C** 3D structural model of KEAP1 obtained from the PDB database. **D** Predicted interface of one of the complexes formed by the binding of TMEM160 and KEAP1. **E** IF staining of Myc-TMEM160 and KEAP1 in BGC-823 and HGC-27 cells. **F**, **G** IP experiments performed on HEK-293T cells co-transfected with Myc-TMEM160 and HA-KEAP1 plasmids; detection of the exogenous interaction between TMEM160 and KEAP1 by WB. **H** Endogenous IP experiments performed in BGC-823 and HGC-27 cells; detection of the endogenous interaction between TMEM160 and KEAP1 by WB. **I** Confirmation of the direct binding between His-KEAP1 and GST-TMEM160 via GST-pull down assay. **J** Schematic diagram of wild-type KEAP1 plasmid and various truncated mutant plasmids. **K** Co-transfection of HEK-293T cells with Myc-TMEM160, Flag-KEAP1 WT plasmid, and various truncated mutant plasmids as indicated; IP performed using an anti-Flag antibody; detection of the interaction between TMEM160 and KEAP1 KELCH domain by WB. Independent biological experiments were repeated at least three times.

To further investigate the potential mechanism by which TMEM160 protects GC cells from ferroptosis, we used the BioGRID and GeneMANIA online databases to search for potential TMEM160 substrates involved in ferroptosis resistance and found that TMEM160 may interact with KEAP1 (Fig. 3A, Supplementary Fig. 3A, and Supplementary Table 7). Given that the KEAP1/NRF2 antioxidant signaling pathway regulates ROS and iron metabolism-related genes and is a key negative regulator of ferroptosis in tumor cells [16, 18, 20], we hypothesized that TMEM160 regulates ferroptosis in GC cells via the KEAP1/NRF2 pathway. To verify this possibility, we used the Z-DOCK tool to perform molecular docking of the three-dimensional structures of TMEM160 and KEAP1. The results showed that TMEM160 formed a spatially stable protein complex with KEAP1 (Fig. 3B–D). IF experiments showed significant co-localization of TMEM160 and KEAP1 in GC cells (Fig. 3E). To further confirm the interaction between TMEM160 and KEAP1, HEK-293T cells were co-transfected with HA-KEAP1 and Myc-TMEM160 plasmids. Co-IP experiments revealed an interaction between TMEM160 and KEAP1 (Fig. 3F, G). Additionally, endogenous Co-IP experiments with BGC-823 and HGC-27 cells confirmed the interaction between TMEM160 and KEAP1 (Fig. 3H). Furthermore, GST pull-down experiments demonstrated that TMEM160 could directly bind to the KEAP1 protein in vitro (Fig. 3I).

To dissect the functional domains involved in the TMEM160-KEAP1 interaction, we designed a series of truncated KEAP1 mutants (Fig. 3J). Exogenous co-IP experiments revealed that the KELCH domain (and not the BTB or IVR domains) of KEAP1 mediated this interaction with TMEM160 (Fig. 3K). In summary, these observations suggest that the interaction between TMEM160 and the KELCH domain of KEAP1 may induce the malignant progression of GC.

### TMEM160 promotes K48-linked ubiquitination and degradation of KEAP1 protein

**Fig. 4:**
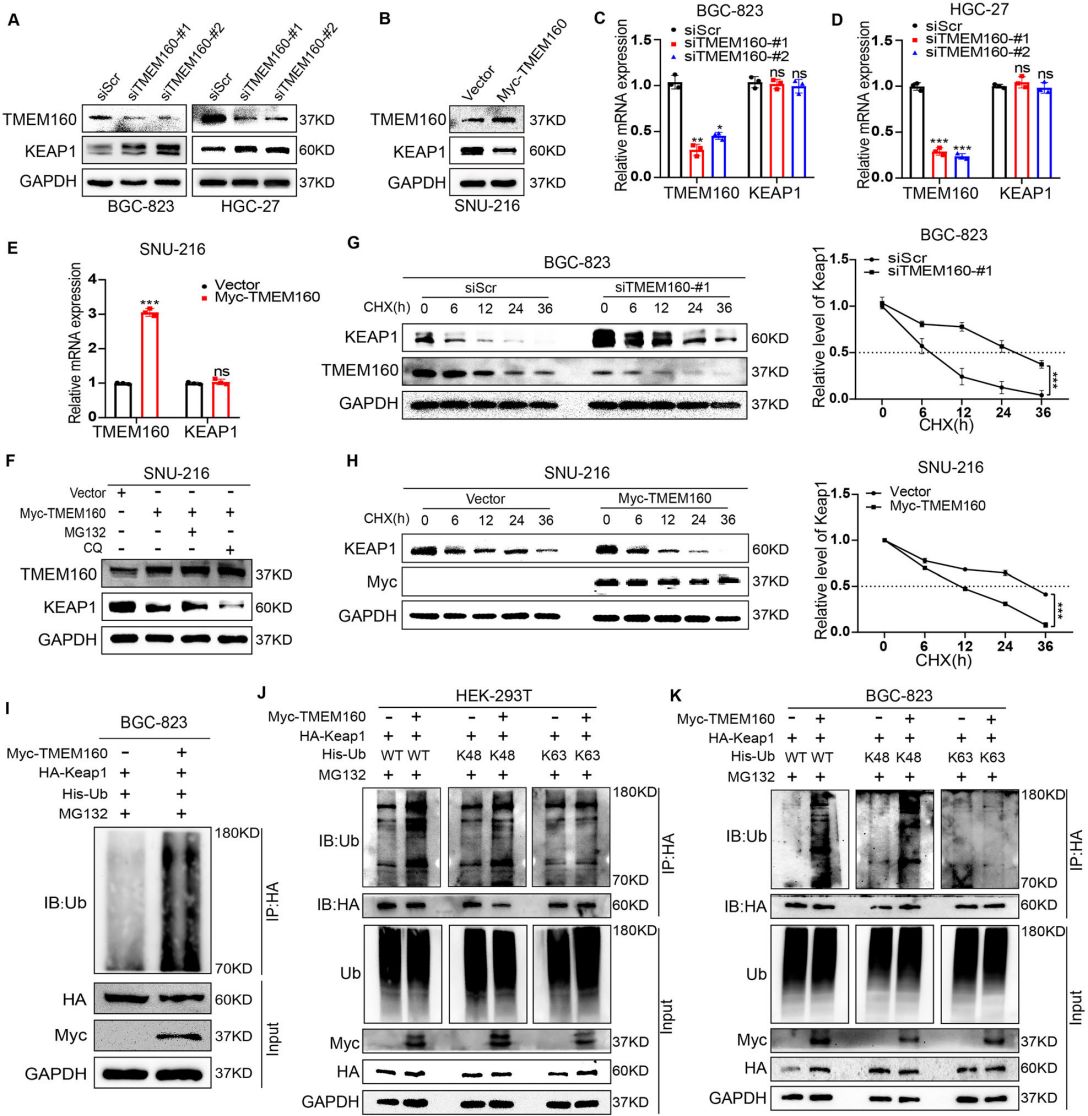
TMEM160 promoted K48-linked ubiquitination and degradation of KEAP1. **A**, **C**, and **D** Downregulation of TMEM160 in BGC-823 and HGC-27 cells using siRNA; detection of KEAP1 protein expression by WB and RT-qPCR. **B**, **E** Upregulation of TMEM160 in SNU-216 cells using Myc-TMEM160 plasmids; detection of KEAP1 protein expression by WB and RT-qCR. **F** Treatment of SNU-216 cells transfected with Myc-TMEM160 or Vector plasmid with MG132 and CQ; detection of changes in KEAP1 protein. **G** Treatment of BGC-823 cells transfected with siScr and siTMEM160-#1 with CHX; detection of changes in KEAP1 protein by WB. **H** Treatment of SNU-216 cells transfected with Vector and Myc-TMEM160 plasmid with CHX; detection of changes in KEAP1 protein by WB. **I** Co-transfection of BGC-823 cells with HA-KEAP1, Myc-TMEM160, and His-Ub plasmids; IP performed using the indicated antibodies; detection of the effect on KEAP1 protein ubiquitination. **J**, **K** Co-transfection of HEK-293T and BGC-823 cells with HA-KEAP1, Myc-TMEM160, and His-WT-Ub or its mutant (His-K48-Ub or His-K63-Ub) plasmids; IP performed using the indicated antibodies; detection of the effect on KEAP1 protein ubiquitination. Independent biological experiments were repeated at least thrice, and the data are presented as the means ± SDs. Statistical differences are indicated by *p*-values, ^*^p < 0.05, ^**^p < 0.01, and ^***^p < 0.001.

Next, we investigated whether TMEM160 regulates the mRNA or protein levels of KEAP1 through interaction. To this end, we established GC cells with upregulated and downregulated TMEM160 expression and assessed KEAP1 expression levels using RT-qPCR and WB. The results showed that TMEM160 overexpression reduced KEAP1 protein levels without altering KEAP1 mRNA levels, whereas TMEM160 knockdown had the opposite effect (Fig. 4A–E). Next, we evaluated the effect of TMEM160 on the stability of the KEAP1 protein. Half-life experiments revealed that TMEM160 knockdown stabilized KEAP1 protein levels in BGC-823 cells, whereas TMEM160 overexpression had the opposite effect (Fig. 4G, H). Notably, TMEM160-promoted KEAP1 degradation could be rescued by the proteasome inhibitor MG132 but not by the autophagy inhibitor CQ (Fig. 4F), suggesting that TMEM160 may degrade KEAP1 through the ubiquitin-proteasome pathway.

To investigate whether TMEM160 affects KEAP1 ubiquitination, we performed ubiquitination assays using BGC-823 cells. As expected, TMEM160 overexpression increased KEAP1 ubiquitination in GC cells (Fig. 4I). Moreover, we constructed K48 and K63 ubiquitin mutant plasmids by retaining two lysine residues on the ubiquitin molecule while mutating all other lysine residues to arginine. Ubiquitination experiments in HEK-293T and BGC-823 cells revealed that TMEM160 overexpression promoted K48-linked polyubiquitination of KEAP1 but did not promote K63-linked ubiquitination (Fig. 4J, K). In summary, these results suggest that TMEM160 disrupted KEAP1 stability by promoting K48-linked polyubiquitination and degradation via interaction with the KELCH domain of KEAP1.

### TMEM160 recruits the E3 ligase TRIM37 to promote KEAP1 ubiquitination and degradation

**Fig. 5:**
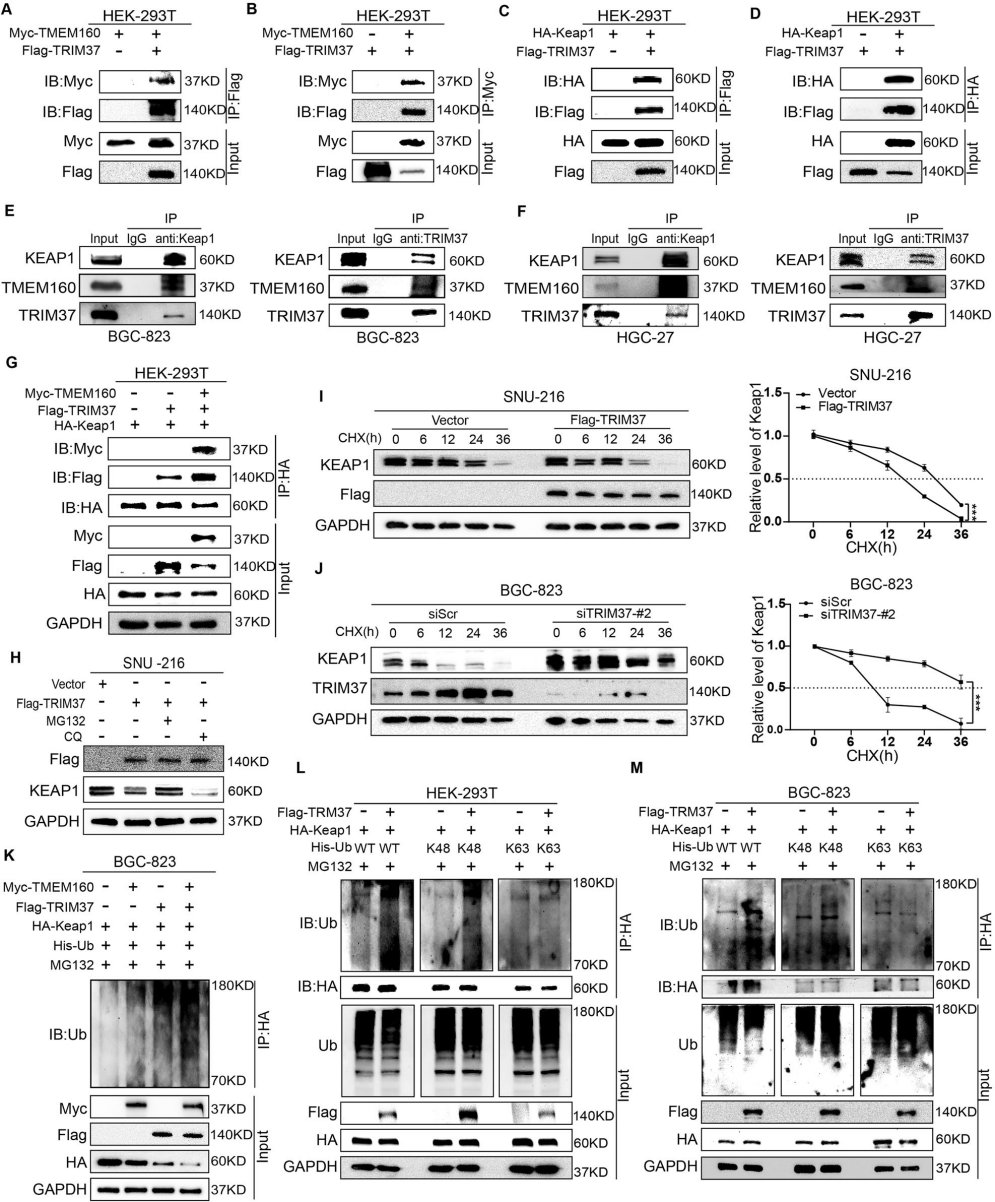
TMEM160 recruited TRIM37 to promote K48-linked ubiquitination and degradation of KEAP1. **A**, **B** IP experiments performed on HEK-293T cells co-transfected with Myc-TMEM160 and Flag-TRIM37 plasmids; detection of the exogenous interaction between TMEM160 and TRIM37 by WB using the indicated antibodies. **C**, **D** IP experiments performed on HEK-293T cells co-transfected with HA-KEAP1 and Flag-TRIM37 plasmids; detection of the exogenous interaction between KEAP1 and TRIM37 by WB using the indicated antibodies. **E**, **F** Endogenous Co-IP experiments performed in BGC-823 and HGC-27 cells; detection of the endogenous interaction between TMEM160, TRIM37 and KEAP1 by WB. **G** Co-transfection of HEK-293T cells with Myc-TMEM160, Flag-TRIM37 and HA-KEAP1 plasmids as indicated; IP was performed using an anti-HA antibody; detection of the effect of TMEM160 on the interaction between TRIM37 and KEAP1 by WB. **H** Treatment of SNU-216 cells transfected with Flag-TRIM37 or Vector plasmid with MG132 and CQ; detection of changes in KEAP1 protein. **I** Treatment of SNU-216 cells transfected with Vector and Flag-TRIM37 plasmid with CHX; detection of changes in KEAP1 protein by WB. **J** Treatment of BGC-823 cells transfected with siScr and siTRIM37-#2 with CHX; detection of changes in KEAP1 protein by WB. **K** Co-transfection of BGC-823 cells with HA-KEAP1, Myc-TMEM160, Flag-TRIM37, and His-Ub plasmids; IP was performed using the indicated antibodies; detection of the effect on KEAP1 protein ubiquitination. **L**, **M** Co-transfection of HEK-293T and BGC-823 cells with HA-KEAP1, Flag-TRIM37, and His-WT-Ub, or its mutant (His-K48-Ub or His-K63-Ub) plasmids; IP performed using the indicated antibodies; detection of the effect on KEAP1 protein ubiquitination. Independent biological experiments were repeated at least thrice, and the data are presented as the means ± SDs. Statistical differences are indicated by *p*-values, ^*^p < 0.05, ^**^p < 0.01, and ^***^p < 0.001.

The aforementioned findings suggest that TMEM160 promoted the ubiquitination and degradation of KEAP1. However, because the transmembrane protein TMEM160 lacks domains and functions for substrate protein ubiquitination and degradation, we hypothesized that TMEM160 recruits an E3 ligase to promote KEAP1 ubiquitination and degradation. Next, we searched online databases for E3 ligases that interact with TMEM160 and KEAP1 and found that the tripartite motif-containing protein, TRIM37, may be a key mediator (Supplementary Table 8). To verify this hypothesis, we used the Z-DOCK tool to perform molecular docking of the three-dimensional structures of TMEM160 and TRIM37. The results showed that TRIM37 formed a spatially stable protein complex with TMEM160 and KEAP1 (Supplementary Fig. 3B–D). IF experiments showed significant colocalization of TMEM160 and TRIM37 (Supplementary Fig. 3E). TRIM37 interacted with both TMEM160 and KEAP1 via exogenous and endogenous interactions (Fig. 5A–F). Moreover, Myc-TMEM160 overexpression increased the binding between Flag-TRIM37 and HA-KEAP1 (Fig. 5G). Thus, TRIM37 may act as an E3 ligase between TMEM160 and KEAP1, ultimately leading to KEAP1 degradation.

We then examined whether TRIM37 influenced KEAP1 stability and found that TRIM37 overexpression accelerated KEAP1 degradation, which could be reversed by MG132 but not by CQ (Fig. 5H). Additionally, TRIM37 overexpression significantly increased the degradation rate of KEAP1 after CHX treatment, whereas TRIM37 knockdown inhibited its degradation rate (Fig. 5I, J). In GC cells, TRIM37 overexpression significantly increased KEAP1 ubiquitination. TRIM37 overexpression further enhanced TMEM160-mediated KEAP1 ubiquitination (Fig. 5K). Furthermore, TRIM37 overexpression promoted K48-linked polyubiquitination of KEAP1 but did not affect K63-linked ubiquitination (Fig. 5L, M). These results suggest that TMEM160 recruits TRIM37 to promote K48-linked polyubiquitination and degradation of KEAP1.

### TMEM160 activates NRF2/GPX4/SLC7A11 axis to inhibit ferroptosis and enhance chemoresistance in an NRF2-dependent manner

**Fig. 6:**
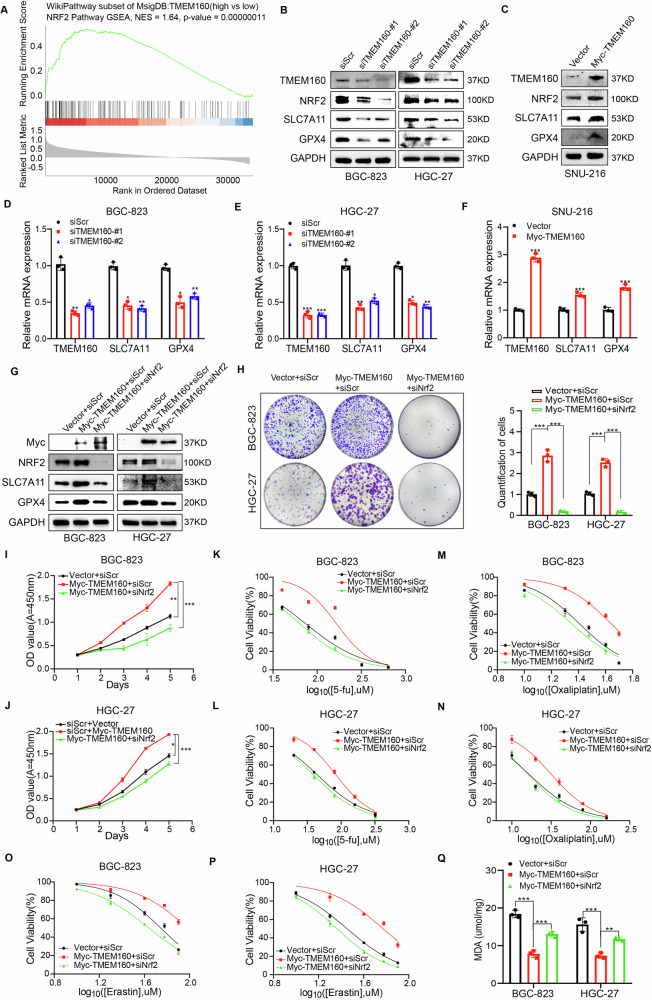
TMEM160 activated NRF2/GPX4/SLC7A11 axis to inhibit ferroptosis and enhance chemoresistance in an NRF2-dependent manner. **A** GESA analysis using the WikiPathways subset of the MsigDB database on GC. **B**, **D**, and **E** Downregulation of TMEM160 in BGC-823 and HGC-27 cells using siRNA; detection of GPX4 and SLC7A11 at the protein and mRNA levels by WB and RT-qPCR. **C**, **F** Upregulation of TMEM160 in SNU-216 cells using Myc-TMEM160 plasmids; detection of GPX4 and SLC7A11 at the protein and mRNA levels by WB and RT-qPCR. Co-transfection of BGC-823 and HGC-27 cells with siScr, siNRF2, vector, and Myc-TMEM160 plasmids as indicated. **G** Detection of NRF2 and its downstream target genes at the protein level by WB. **H** Colony formation assay was performed to assess cell colony-forming ability. **I**, **J** CCK-8 assays were performed to assess cell proliferation ability. **K–N** Toxic proliferation assays were performed to assess sensitivity to chemotherapy drugs (5-fu and Oxaliplatin). **O**, **P** Toxic proliferation assays were performed to assess sensitivity to erastin. **Q** MDA levels detected using an MDA assay kit. Independent biological experiments were repeated at least thrice, and the data are presented as the means ± SDs. Statistical differences are indicated by *p*-values of ^*^p < 0.05, ^**^p < 0.01, and ^***^p < 0.001.

Interestingly, the Gene Set enrichment analysis (GSEA) based on the MsigDB database revealed a positive correlation between TMEM160 and the NRF2 pathway (Fig. 6A and Supplementary Fig. 4A). The NRF2 pathway transcriptionally regulates the expression of *SLC7A11* and *GPX4* to inhibit ferroptosis in cancer cells [16]. In order to determine whether TMEM160 regulates the expression of GPX4 and SLC7A11 through NRF2 to inhibit ferroptosis, we performed RT-qPCR and WB experiments and found that inhibiting TMEM160 reduced NRF2 protein level, whereas it overexpression had the opposite effect, but TMEM160 did not affect NRF2 mRNA expression (Fig. 6B–C and Supplementary Fig. 4B–D). Moreover, TMEM160 downregulation in GC cells reduced the protein and mRNA levels of NRF2 downstream target genes, *GPX4* and *SLC7A11*, whereas its overexpression had the opposite effect (Fig. 6B–F). These results suggest that TMEM160 activated NRF2 and promoted the expression of downstream target genes *GPX4* and *SLC7A11*, thereby protecting GC cells from ferroptosis.

Considering that we demonstrated the inhibition of ferroptosis by TMEM160 in GC cells and the activation of the NRF2/GPX4/SLC7A11 axis, we further investigated whether TMEM160 inhibited ferroptosis in an NRF2-dependent manner. We knocked down *NRF2* in BGC-823 and HGC-27 cells with TMEM160 overexpression and found that TMEM160 overexpression inhibited erastin-induced ferroptosis, whereas *NRF2* knockdown significantly restored the sensitivity of GC cells to erastin (Fig. 6G, O, and P and Supplementary Table 6). Moreover, *NRF2* knockdown reversed the inhibition of lipid peroxidation attributed to TMEM160 overexpression (Fig. 6Q). Additionally, *NRF2* knockdown significantly weakened the protective effect of TMEM160 on GC cell proliferation and increased the sensitivity of GC cells to chemotherapeutic drugs (Fig. 6H–N and Supplementary Table 6). In summary, these results suggest that TMEM160 inhibited ferroptosis and promoted chemoresistance in GC cells in a partial NRF2-dependent manner.

Collectively, TMEM160 activates NRF2/GPX4/SLC7A11 axis and inhibits ferroptosis in an NRF2-dependent manner, thereby inducing chemoresistance in GC cells.

### TMEM160 promotes tumor growth and chemoresistance in vivo

**Fig. 7:**
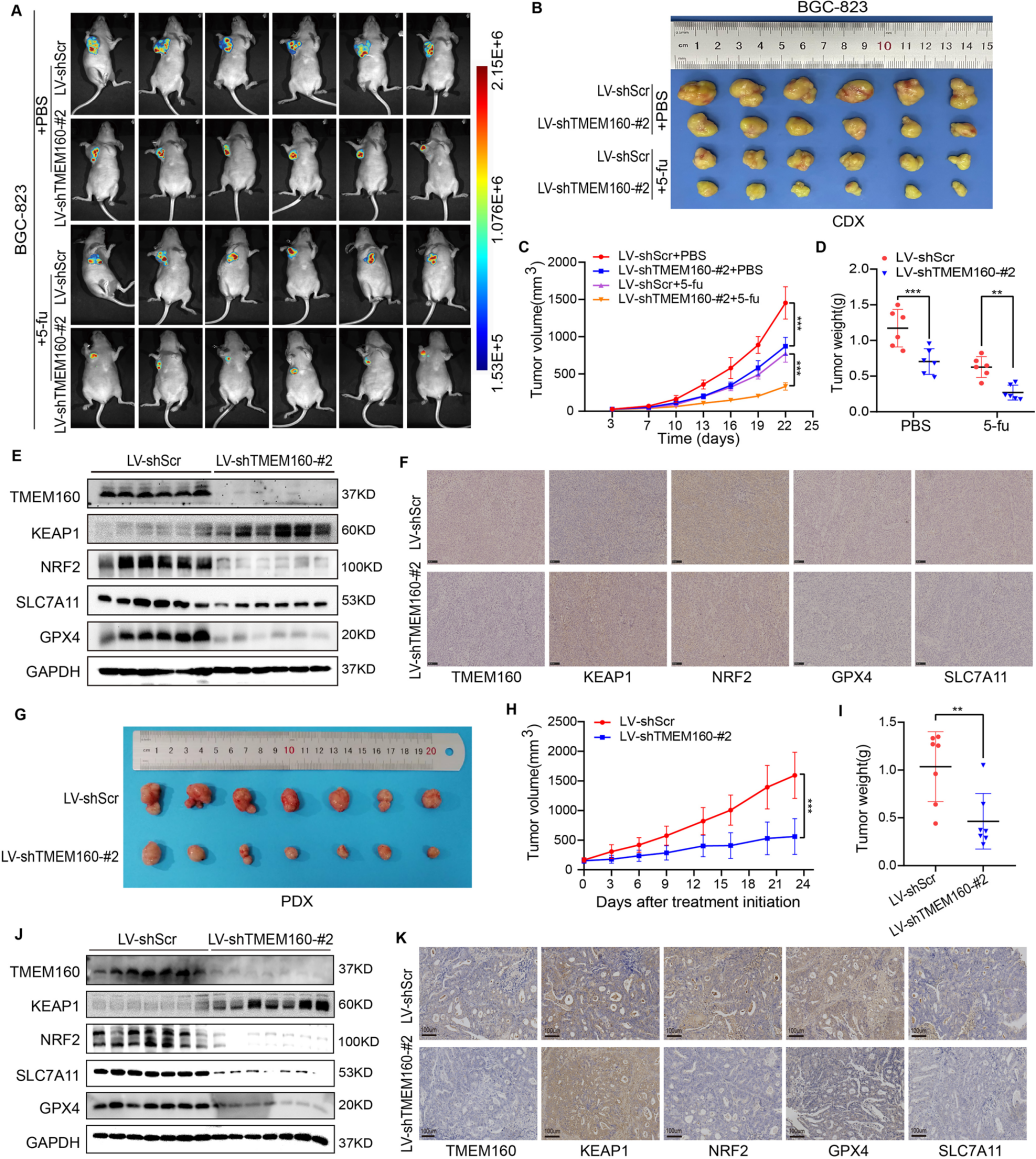
Targeting TMEM160 inhibited GC growth and promoted chemotherapy sensitivity in vivo. BGC-823 cells stably transfected with the designated lentiviral vectors were subcutaneously implanted into female BALB/c nude mice to establish xenograft models, followed by the intraperitoneal injections of 5-fu and PBS. **A** In vivo imaging of tumors (*n* = 6 per group). **B** Macroscopic images of tumors (*n* = 6 per group). **C** Growth curves of tumor volume (*n* = 6 per group). **D** Tumor weight (*n* = 6 per group). **E** WB detected the expression of TMEM160, KEAP1, NRF2, SLC7A11, and GPX4 in cell-derived xenografts. **F** Representative IHC images of TMEM160, KEAP1, NRF2, SLC7A11, and GPX4 in cell-derived xenografts, scale bar: 100 μm. Fresh tissues obtained from patients with GC were used to establish PDX models, and LV-shScr and LV-shTMEM160-#2 lentiviruses were injected into the tumors. **G** Macroscopic images of the tumors (*n* = 7 per group). **H** Growth curves of tumor volume (*n* = 7 per group). **I** Tumor weight (*n* = 7 per group). **J** WB detected the expression of TMEM160, KEAP1, NRF2, SLC7A11, and GPX4 in PDXs. **K** Representative IHC images of TMEM160, KEAP1, NRF2, SLC7A11, and GPX4 in PDXs, scale bar: 100 μm. Data are presented as mean ± SDs, and statistical differences are indicated by *p*-values of ^**^p < 0.01, and ^***^p < 0.001.

To confirm the aforementioned findings, we investigated the effects of TMEM160 expression on GC growth and chemoresistance in vivo. We stably transfected BGC-823 and HGC-27 cells with a lentivirus to knock down TMEM160 (Supplementary Fig. 5A, B) and subcutaneously implanted LV-shScr and LV-shTMEM160-#2-infected cells into BALB/c nude mice. We found that the tumor growth was significantly inhibited in the LV-shTMEM160-#2 group compared to that in the control group (Fig. 7A, B and Supplementary Fig. 5C, D, G, and H), and tumor volume and weight were significantly reduced (Fig. 7C, D, Supplementary Fig. 5E, F, I, and J). Similar results were obtained with the GC PDX model, which we successfully established using primary tumor cells derived from a patient with GC. After intratumoral injection of LV-shTMEM160-#2 lentivirus, tumor growth was significantly inhibited (Fig. 7G). Compared to the control group, the tumor growth rate was significantly slower, and the tumor volume and mass were significantly reduced in the LV-shTMEM160-#2 group (Fig. 7H, I). Moreover, in the CDX model, intraperitoneal injection of 5-fu further inhibited tumor growth in the LV-shTMEM160-#2 group (Fig. 7A, B and Supplementary Fig. 5G, H).

WB and IHC analysis of subcutaneous tumor tissues in the CDX and PDX models showed that TMEM160 knockdown reduced the expression of NRF2 and its target genes, *GPX4* and *SLC7A11* (Fig. 7E, F, J, and K). These results further confirmed that TMEM160 promoted GC growth and reduced chemotherapy sensitivity in vivo by activating the NRF2/GPX4/SLC7A11 axis.

### TMEM160/NRF2 Co-expression as a novel prognostic biomarker in GC

**Fig. 8:**
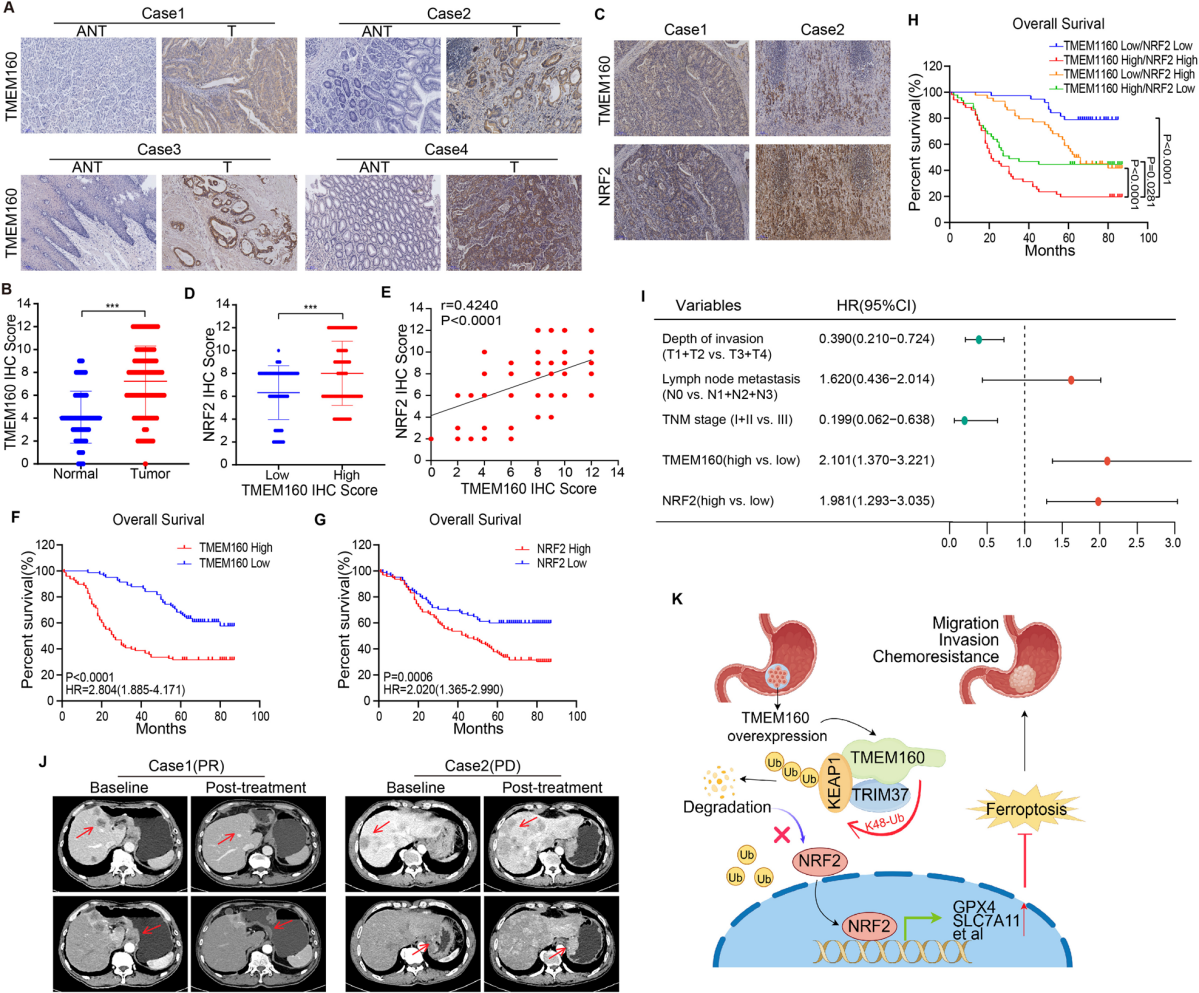
TMEM160 and NRF2 were overexpressed in GC tissues, and their co-overexpression predicted poor prognosis in GC. **A**, **C** Representative IHC images of TMEM160 and NRF2 expression in clinical gastric tumor tissues and adjacent normal tissues, scale bar: 100 μm. **B** TMEM160 IHC scores in clinical GC tissues and adjacent normal tissues. **D** NRF2 IHC scores in clinical gastric tumor tissues with high or low TMEM160 IHC scores. **E** Spearman correlation analysis of TMEM160 and NRF2 IHC scores in gastric tumor tissues. **F**–**H** Kaplan–Meier survival analysis based on 180 GC patients who underwent surgical treatment. **I** Multivariate Cox regression analysis based on 180 GC patients who underwent surgical treatment. **J** The CT images of two patients with advanced GC before and after treatment. The first patient had low expression levels of TMEM160 and NRF2 (Case 1), whereas the second patient had high expression levels (Case 2). Data are presented as mean ± SDs, statistical differences are indicated by *p*-values. ^***^p < 0.001. **K** Schematic diagram of TMEM160 function in GC cells.TMEM160 recruited the E3 ligase TRIM37 to promote K48-linked ubiquitination and degradation of KEAP1, thereby activating NRF2 and transcriptionally upregulating the target genes *GPX4* and *SLC7A11* to inhibit ferroptosis and induce chemoresistance in GC cells. The figure was drawn using Figdraw.

Through TCGA database analysis, we identified TMEM160 as a pan-cancer upregulated gene, with significant mRNA elevation in GC and other solid tumors (Supplementary Fig. 6A, B). In our institutional cohort of 180 GC patients who underwent surgical treatment (clinicopathological characteristics in Supplementary Table 9), IHC analysis revealed concurrent overexpression of TMEM160 and NRF2 in tumor versus adjacent normal tissues (Fig. 8A, B and Supplementary Fig. 6C). Notably, TMEM160 expression levels showed a strong positive correlation with NRF2 (Spearman *r* = 0.4240, *p* < 0.0001, Fig. 8C–E), suggesting potential functional interplay. Survival analysis demonstrated that high TMEM160 expression was associated with significantly reduced overall survival (median OS = 26 vs undefined months, *p* < 0.0001; Fig. 8F), a finding independently validated in the TCGA STAD cohort(Supplementary Fig. 6D, E). Importantly, dual-high TMEM160/NRF2 expression defined a distinct subgroup with the poorest prognosis (median OS = 21 months, *p* < 0.0001, Fig. 8H). Univariate and multivariate analysis confirmed both markers as independent prognostic factors (TMEM160: HR = 2.101, 95% CI 1.370–3.221, *p* = 0.001; NRF2: HR = 1.981, 95% CI 1.293–3.035, *p* = 0.002; Fig. 8I, Supplementary Fig. 6F, and Supplementary Table 10).

To further elucidate the clinical significance of TMEM160 in advanced GC, we also performed IHC analysis on biopsy tissues from 89 patients with advanced GC. In advanced GC patients(clinicopathological characteristics in Supplementary Table 11), the TMEM160/NRF2 signature demonstrated predictive value for treatment response. Dual-high expressors showed rapid disease progression following first-line chemoimmunotherapy (Fig. 8J Case2), contrasting with marked tumor regression in dual-low cases (Fig. 8J Case1). Correspondingly, dual-high patients exhibited significantly shorter progression-free survival (median PFS = 3 vs 7 months in dual-low patients, *p* < 0.0001, Supplementary Fig. 6G, H), with both markers independently predicting treatment resistance (TMEM160: HR = 3.550, 95% CI: 1.926–6.441, *p* < 0.001; NRF2: HR = 2.587, 95% CI: 1.424–4.701, *p* = 0.002; Supplementary Fig. 6I, J and Supplementary Table 12).

These findings establish the TMEM160/NRF2 axis as a novel prognostic and predictive biomarker system in GC, with dual-high expression identifying a distinct subset of patients with aggressive disease biology and treatment resistance.

## Discussion

To the best of our knowledge, this is the first study to confirm that TMEM160 is a critical regulator of ferroptosis inhibition in GC. TMEM160 promoted GC cells proliferation, invasion, migration, and chemoresistance by recruiting the E3 ligase TRIM37 to promote K48-linked ubiquitination and degradation of KEAP1, thereby activating the NRF2/GPX4/SLC7A11 axis and inhibiting ferroptosis (Fig. 8K). The findings of our study revealed the mechanism by which TMEM160 promotes GC growth and chemoresistance, thereby providing a promising novel therapeutic target for GC.

### TMEM160 promotes ferroptosis resistance and drives tumor growth and chemoresistance in GC

Accumulating evidence suggests that ferroptosis serves as a natural tumor-suppressive mechanism, functioning through interactions with multiple tumor suppressor genes, such as *P53, KEAP1, ARF*, and *MLL4* [17, 31–33]. Targeting ferroptosis-related genes to induce ferroptosis has emerged as an effective novel therapeutic strategy for counteracting tumor progression and chemoresistance [12, 34, 35]. Our study identified a critical role for TMEM160 in modulating ferroptosis in GC cells. The correlation between TMEM160 and GPX4, a key ferroptosis inhibitor [11, 12, 31, 36], indicates that TMEM160 is deeply involved in the regulation of ferroptosis in GC. We further demonstrated that knockdown of TMEM160 significantly increased erastin-induced death in GC cells, which was reversed by the ferroptosis inhibitor Ferr-1. Ferroptosis is characterized by the accumulation of iron-dependent lipid peroxides and membrane rupture [31, 37, 38], the phenotypic changes observed, such as mitochondrial shrinkage, increased membrane density, elevated lipid peroxidation and Fe^2+^ levels, and reduced GSH levels, provide compelling evidence that TMEM160 functions as a pivotal negative regulator of ferroptosis in GC cells.

Abnormal resistance to ferroptosis can promote malignant growth and chemoresistance in cancer cells, posing a significant obstacle to effective treatment [39]. Here, we observed that the downregulation of TMEM160 in GC cells led to inhibited proliferation, reduced invasion and migration, and enhanced sensitivity to chemotherapy drugs. These findings suggest that TMEM160 not only protects GC cells from ferroptosis but also contributes to malignant progression by fostering a resistance to ferroptosis, a characteristic feature of many aggressive cancers. In vivo experiments further supported this notion, as targeting TMEM160 expression in both CDX and PDX models significantly suppressed xenograft growth. Taken together, these findings suggest, for the first time, that TMEM160 is a key player in GC progression and therapeutic resistance, and its inhibition could provide a therapeutic benefit.

### TMEM160 promotes ferroptosis resistance and chemoresistance in GC via the TRIM37-KEAP1/NRF2 axis

Our study further delves into the mechanism by which TMEM160 regulates ferroptosis resistance and chemoresistance in GC. Aberrant activation of the KEAP1/NRF2 signaling pathway is closely associated with tumor cell development and chemoresistance [18, 40]. As such, inhibiting the NRF2 signaling pathway has recently been considered a promising approach to curbing tumor growth and overcoming chemoresistance [19–21, 41]. Our work is the first to establish a direct interaction between TMEM160 and KEAP1, providing important new insights into how TMEM160 regulates the KEAP1/NRF2 pathway. Notably, our findings suggest that TMEM160 does not affect KEAP1 mRNA levels but instead modulates its protein stability through post-transcriptional mechanisms, likely involving protein degradation. Furthermore, our study found that TMEM160 promoted KEAP1 protein degradation via the ubiquitin-proteasome pathway. This novel aspect of TMEM160 function presents an interesting point for future research, as it suggests that TMEM160 may exert its effects through fine-tuned regulation of ubiquitination degradation for KEAP1, rather than transcriptional regulation.

The discovery of TRIM37 as an intermediary E3 ligase linking TMEM160 to KEAP1 degradation represents a significant advancement in our understanding of the molecular mechanisms underlying ferroptosis resistance.TRIM37 is a tripartite motif ubiquitin ligase characterized by a RING, B-box, and coiled-coil (RBCC) ubiquitin ligase domain, along with a TRAF domain [42], it promotes tumorigenesis via various mechanisms [43, 44]. We found that the interaction between TMEM160 and TRIM37 could induce K48-linked polyubiquitination and degradation of the KEAP1 protein. This degradation reduces KEAP1 levels, thereby stabilizing NRF2, which in turn activates the transcription of downstream genes, such as *GPX4* and *SLC7A11*, that are directly involved in ferroptosis resistance. This TMEM160-TRIM37-KEAP1/NRF2 axis not only highlights a critical regulatory pathway in GC but also suggests potential therapeutic targets to enhance the efficacy of treatments by overcoming ferroptosis resistance.

### High expression of TMEM160 is associated with poor prognosis in GC, and targeting TMEM160 enhances chemotherapy sensitivity

Effective prognostic biomarkers for GC are lacking in clinical practice. In this study, we found that TMEM160 was markedly overexpressed in clinical gastric tumor tissues, and its elevated expression was significantly associated with poor prognosis. These findings suggest that TMEM160 could serve as an independent prognostic factor for GC patients, offering valuable insights into patient outcomes and therapeutic response. Additionally, we observed a strong positive correlation between TMEM160 and NRF2 expression in GC, with patients co-overexpressing TMEM160 and NRF2 exhibiting poor prognosis and reduced therapeutic efficacy. This relationship further emphasizes the clinical relevance of TMEM160 and NRF2 as reliable prognostic biomarkers for GC.

From a therapeutic perspective, our study demonstrates that targeting TMEM160 in combination with conventional chemotherapy drugs such as 5-fu significantly enhances the sensitivity of GC cells to chemotherapy and inhibits tumor growth both in vitro and in vivo. This finding is particularly exciting because it suggests that the development of small-molecule inhibitors targeting TMEM160 could serve as a novel adjunctive treatment strategy, potentially improving the outcomes of chemotherapy in GC patients.

## Conclusion and future directions

In summary, this study systematically confirmed that the TMEM160-TRIM37-KEAP1/NRF2 axis significantly influenced GC progression and chemoresistance. We identified TMEM160 as a crucial negative regulator of ferroptosis and highlight its potential as a therapeutic target for overcoming chemoresistance in GC. The identification of TMEM160 as a novel oncogenic factor in GC opens new possibilities for targeted therapies aimed at restoring ferroptosis sensitivity in GC cells. Future studies should focus on the development of small-molecule inhibitors targeting TMEM160 and further elucidate the molecular pathways underlying its regulation of ferroptosis and chemoresistance. Moreover, exploring the clinical implications of TMEM160 as a prognostic biomarker will be critical for translating these findings into therapeutic strategies that can improve patient outcomes in GC.

## Supplementary information

Supplementary Figures and Tables

Supplement 1

Supplement 2

Raw blots (the raw blots image)

## Data Availability

The datasets used and/or analyzed during the current study are available from the corresponding author upon reasonable request.

## References

[1] Sung H, Ferlay J, Siegel RL, Laversanne M, Soerjomataram I, Jemal A, et al. Global cancer statistics 2020: GLOBOCAN estimates of incidence and mortality worldwide for 36 cancers in 185 countries. CA Cancer J Clin. 2021;71:209–49. 33538338 10.3322/caac.21660

[2] Zheng RS, Chen R, Han BF, Wang SM, Li L, Sun KX, et al. Cancer incidence and mortality in China, 2022. Zhonghua Zhong Liu Za Zhi. 2024;46:221–31. 38468501 10.3760/cma.j.cn112152-20240119-00035

[3] Smyth EC, Nilsson M, Grabsch HI, van Grieken NC, Lordick F. Gastric cancer. Lancet. 2020;396:635–48. 32861308 10.1016/S0140-6736(20)31288-5

[4] Noh SH, Park SR, Yang HK, Chung HC, Chung IJ, Kim SW, et al. Adjuvant capecitabine plus oxaliplatin for gastric cancer after D2 gastrectomy (CLASSIC): 5-year follow-up of an open-label, randomised phase 3 trial. Lancet Oncol. 2014;15:1389–96. 25439693 10.1016/S1470-2045(14)70473-5

[5] Joshi SS, Badgwell BD. Current treatment and recent progress in gastric cancer. CA Cancer J Clin. 2021;71:264–79. 33592120 10.3322/caac.21657 PMC9927927

[6] Dixon SJ, Lemberg KM, Lamprecht MR, Skouta R, Zaitsev EM, Gleason CE, et al. Ferroptosis: an iron-dependent form of nonapoptotic cell death. Cell. 2012;149:1060–72. 22632970 10.1016/j.cell.2012.03.042 PMC3367386

[7] Stockwell BR, Friedmann Angeli JP, Bayir H, Bush AI, Conrad M, Dixon SJ, et al. Ferroptosis: a regulated cell death nexus linking metabolism. Redox Biol, Dis Cell. 2017;171:273–85. 10.1016/j.cell.2017.09.021 PMC5685180 28985560

[8] Tang D, Chen X, Kang R, Kroemer G. Ferroptosis: molecular mechanisms and health implications. Cell Res. 2021;31:107–25. 33268902 10.1038/s41422-020-00441-1 PMC8026611

[9] Jiang X, Stockwell BR, Conrad M. Ferroptosis: mechanisms, biology and role in disease. Nat Rev Mol Cell Biol. 2021;22:266–82. 33495651 10.1038/s41580-020-00324-8 PMC8142022

[10] Koppula P, Zhuang L, Gan B. Cystine transporter SLC7A11/xCT in cancer: ferroptosis, nutrient dependency, and cancer therapy. Protein Cell. 2021;12:599–620. 33000412 10.1007/s13238-020-00789-5 PMC8310547

[11] Yang WS, SriRamaratnam R, Welsch ME, Shimada K, Skouta R, Viswanathan VS, et al. Regulation of ferroptotic cancer cell death by GPX4. Cell. 2014;156:317–31. 24439385 10.1016/j.cell.2013.12.010 PMC4076414

[12] Zou Y, Palte MJ, Deik AA, Li H, Eaton JK, Wang W, et al. A GPX4-dependent cancer cell state underlies the clear-cell morphology and confers sensitivity to ferroptosis. Nat Commun. 2019;10:1617. 30962421 10.1038/s41467-019-09277-9 PMC6453886

[13] Hayes JD, Dinkova-Kostova AT, Tew KD. Oxidative stress in cancer. Cancer Cell. 2020;38:167–97. 32649885 10.1016/j.ccell.2020.06.001 PMC7439808

[14] Cuadrado A, Rojo AI, Wells G, Hayes JD, Cousin SP, Rumsey WL, et al. Therapeutic targeting of the NRF2 and KEAP1 partnership in chronic diseases. Nat Rev Drug Discov. 2019;18:295–317. 30610225 10.1038/s41573-018-0008-x

[15] Wang J, Lu Q, Cai J, Wang Y, Lai X, Qiu Y, et al. Nestin regulates cellular redox homeostasis in lung cancer through the Keap1-Nrf2 feedback loop. Nat Commun. 2019;10:5043. 31695040 10.1038/s41467-019-12925-9 PMC6834667

[16] Fan Z, Wirth AK, Chen D, Wruck CJ, Rauh M, Buchfelder M, et al. Nrf2-Keap1 pathway promotes cell proliferation and diminishes ferroptosis. Oncogenesis. 2017;6:e371. 28805788 10.1038/oncsis.2017.65 PMC5608917

[17] Mou Y, Wang J, Wu J, He D, Zhang C, Duan C, et al. Ferroptosis, a new form of cell death: opportunities and challenges in cancer. J Hematol Oncol. 2019;12:34. 30925886 10.1186/s13045-019-0720-y PMC6441206

[18] Han Y, Gao X, Wu N, Jin Y, Zhou H, Wang W, et al. Long noncoding RNA LINC00239 inhibits ferroptosis in colorectal cancer by binding to Keap1 to stabilize Nrf2. Cell Death Dis. 2022;13:742. 36038548 10.1038/s41419-022-05192-y PMC9424287

[19] Zhang Q, Zhang ZY, Du H, Li SZ, Tu R, Jia YF, et al. DUB3 deubiquitinates and stabilizes NRF2 in chemotherapy resistance of colorectal cancer. Cell Death Differ. 2019;26:2300–13. 30778200 10.1038/s41418-019-0303-z PMC6889501

[20] Ge W, Zhao K, Wang X, Li H, Yu M, He M, et al. iASPP Is an antioxidative factor and drives cancer growth and drug resistance by competing with Nrf2 for Keap1 binding. Cancer Cell. 2017;32:561–73.e6. 29033244 10.1016/j.ccell.2017.09.008

[21] Zhou Y, Chen Y, Shi Y, Wu L, Tan Y, Li T, et al. FAM117B promotes gastric cancer growth and drug resistance by targeting the KEAP1/NRF2 signaling pathway. J Clin Invest. 2023;133:e158705. 10.1172/JCI158705 PMC9888386 36719368

[22] Liu JZ, Hu YL, Feng Y, Jiang Y, Guo YB, Liu YF, et al. BDH2 triggers ROS-induced cell death and autophagy by promoting Nrf2 ubiquitination in gastric cancer. J Exp Clin Cancer Res. 2020;39:123. 32605589 10.1186/s13046-020-01620-z PMC7325376

[23] Yoo NJ, Kim HR, Kim YR, An CH, Lee SH. Somatic mutations of the KEAP1 gene in common solid cancers. Histopathology. 2012;60:943–52. 22348534 10.1111/j.1365-2559.2012.04178.x

[24] Dai X, Wu Z, Ruan R, Chen J, Huang C, Lei W, et al. TMEM160 promotes tumor immune evasion and radiotherapy resistance via PD-L1 binding in colorectal cancer. Cell Commun Signal. 2024;22:168. 38454413 10.1186/s12964-024-01541-w PMC10921666

[25] Yamashita K, Haraguchi M, Yano M. Knockdown of TMEM160 leads to an increase in reactive oxygen species generation and the induction of the mitochondrial unfolded protein response. FEBS Open Biol. 2022;12:2179–90. 10.1002/2211-5463.13496 PMC9714381 36217717

[26] Li X, Zhong H, Shi Q, Ruan R, Huang C, Wen Q, et al. YAP1-CPNE3 positive feedback pathway promotes gastric cancer cell progression. Cell Mol Life Sci. 2024;81:143. 38493426 10.1007/s00018-024-05178-3 PMC10944813

[27] Liu Z, Li J, Ding Y, Ma M, Chen J, Lei W, et al. USP49 mediates tumor progression and poor prognosis through a YAP1-dependent feedback loop in gastric cancer. Oncogene. 2022;41:2555–70. 35318441 10.1038/s41388-022-02267-0

[28] Yao Y, Liu Z, Huang S, Huang C, Cao Y, Li L, et al. The E3 ubiquitin ligase, FBXW5, promotes the migration and invasion of gastric cancer through the dysregulation of the Hippo pathway. Cell Death Discov. 2022;8:79. 35210431 10.1038/s41420-022-00868-y PMC8873275

[29] Jiang Y, Mao C, Yang R, Yan B, Shi Y, Liu X, et al. EGLN1/c-Myc induced lymphoid-specific helicase inhibits ferroptosis through lipid metabolic gene expression changes. Theranostics. 2017;7:3293–305. 28900510 10.7150/thno.19988 PMC5595132

[30] Mao C, Wang X, Liu Y, Wang M, Yan B, Jiang Y, et al. A G3BP1-interacting lncRNA promotes ferroptosis and apoptosis in cancer via nuclear sequestration of p53. Cancer Res. 2018;78:3484–96. 29588351 10.1158/0008-5472.CAN-17-3454 PMC8073197

[31] Liang D, Feng Y, Zandkarimi F, Wang H, Zhang Z, Kim J, et al. Ferroptosis surveillance independent of GPX4 and differentially regulated by sex hormones. Cell. 2023;186:2748–64.e22. 37267948 10.1016/j.cell.2023.05.003 PMC10330611

[32] Doll S, Proneth B, Tyurina YY, Panzilius E, Kobayashi S, Ingold I, et al. ACSL4 dictates ferroptosis sensitivity by shaping cellular lipid composition. Nat Chem Biol. 2017;13:91–8. 27842070 10.1038/nchembio.2239 PMC5610546

[33] Mao C, Liu X, Zhang Y, Lei G, Yan Y, Lee H, et al. DHODH-mediated ferroptosis defence is a targetable vulnerability in cancer. Nature. 2021;593:586–90. 33981038 10.1038/s41586-021-03539-7 PMC8895686

[34] Lei G, Zhang Y, Koppula P, Liu X, Zhang J, Lin SH, et al. The role of ferroptosis in ionizing radiation-induced cell death and tumor suppression. Cell Res. 2020;30:146–62. 31949285 10.1038/s41422-019-0263-3 PMC7015061

[35] Wu J, Minikes AM, Gao M, Bian H, Li Y, Stockwell BR, et al. Intercellular interaction dictates cancer cell ferroptosis via NF2-YAP signalling. Nature. 2019;572:402–6. 31341276 10.1038/s41586-019-1426-6 PMC6697195

[36] Ingold I, Berndt C, Schmitt S, Doll S, Poschmann G, Buday K, et al. Selenium utilization by GPX4 is required to prevent hydroperoxide-induced ferroptosis. Cell. 2018;172:409–22.e21. 29290465 10.1016/j.cell.2017.11.048

[37] Bersuker K, Hendricks JM, Li Z, Magtanong L, Ford B, Tang PH, et al. The CoQ oxidoreductase FSP1 acts parallel to GPX4 to inhibit ferroptosis. Nature. 2019;575:688–92. 31634900 10.1038/s41586-019-1705-2 PMC6883167

[38] Doll S, Freitas FP, Shah R, Aldrovandi M, da Silva MC, Ingold I, et al. FSP1 is a glutathione-independent ferroptosis suppressor. Nature. 2019;575:693–8. 31634899 10.1038/s41586-019-1707-0

[39] Lei G, Zhuang L, Gan B. The roles of ferroptosis in cancer: tumor suppression, tumor microenvironment, and therapeutic interventions. Cancer Cell. 2024;42:513–34. 38593779 10.1016/j.ccell.2024.03.011

[40] Homma S, Ishii Y, Morishima Y, Yamadori T, Matsuno Y, Haraguchi N, et al. Nrf2 enhances cell proliferation and resistance to anticancer drugs in human lung cancer. Clin Cancer Res. 2009;15:3423–32. 19417020 10.1158/1078-0432.CCR-08-2822

[41] Adinolfi S, Patinen T, Jawahar Deen A, Pitkänen S, Härkönen J, Kansanen E, et al. The KEAP1-NRF2 pathway: Ttargets for therapy and role in cancer. Redox Biol. 2023;63:102726. 37146513 10.1016/j.redox.2023.102726 PMC10189287

[42] Meitinger F, Ohta M, Lee KY, Watanabe S, Davis RL, Anzola JV, et al. TRIM37 controls cancer-specific vulnerability to PLK4 inhibition. Nature. 2020;585:440–6. 32908304 10.1038/s41586-020-2710-1 PMC7501188

[43] Bhatnagar S, Gazin C, Chamberlain L, Ou J, Zhu X, Tushir JS, et al. TRIM37 is a new histone H2A ubiquitin ligase and breast cancer oncoprotein. Nature. 2014;516:116–20. 25470042 10.1038/nature13955 PMC4269325

[44] Cui G, Gao Z, Chang S, Narwade N, Chen Y, Poudel B, et al. TRIM37 augments AP-2γ transcriptional activity and cellular localization via k63-linked ubiquitination to drive breast cancer progression. Int J Biol Sci. 2022;18:4316–28. 35864973 10.7150/ijbs.69466 PMC9295074

