# Supplementary material for: TMEM160 inhibits KEAP1 to suppress ferroptosis and induce chemoresistance in gastric cancer
Source: Cell Death Dis. 2025 Apr 13;16(1):287. doi: 10.1038/s41419-025-07621-0 (PMC11994801; doi:10.1038/s41419-025-07621-0)
Supplement: Supplementary file 1 — Supplementary Figures and Tables [file 41419_2025_7621_MOESM1_ESM.docx]

**TMEM160 inhibits KEAP1 to suppress ferroptosis and induce chemoresistance in gastric cancer**

Chunye Huang^1,2#^, Qinru Zeng^1,2#^, Jingyi Chen^1,2^, Qin Wen^1,2^, Weilun Jin^1,2^, Xiaofeng Dai^1,2^, Ruiwen Ruan^1,2^, Hongguang Zhong^1,2^, Yang Xia^1,2^, Zhipeng Wu^1,2^, Ruixuan Huang^1,2^, Jianxi Zhang^1,2^,Yangyang Yao^1^, Li Li^1^, Wan Lei^1^, Jianping Xiong*^,1^ and Jun Deng*^,1,2,3^

^1^ Department of Oncology, The First Affiliated Hospital, Jiangxi Medical College, Nanchang University, Nanchang, Jiangxi 33006, China

^2^ Jiangxi Key Laboratory for Individual Cancer Therapy, Nanchang, Jiangxi 330006, China

^3^ Postdoctoral Innovation Practice Base, The First Affiliated Hospital of Nanchang University, Nanchang, Jiangxi 330006, China

**^#^** Co-first authors: Chunye Huang and Qinru Zeng

*Corresponding author: Jun Deng, E-mail address:dengjun19871106@ncu.edu.cn; Jianping Xiong, E-mail address: Jpxiong0630@outlook.com.

**This file includes:**

**Supplementary Figure 1-6**

**Supplementary Figure Legends**

**Supplementary Table 1-13**

**
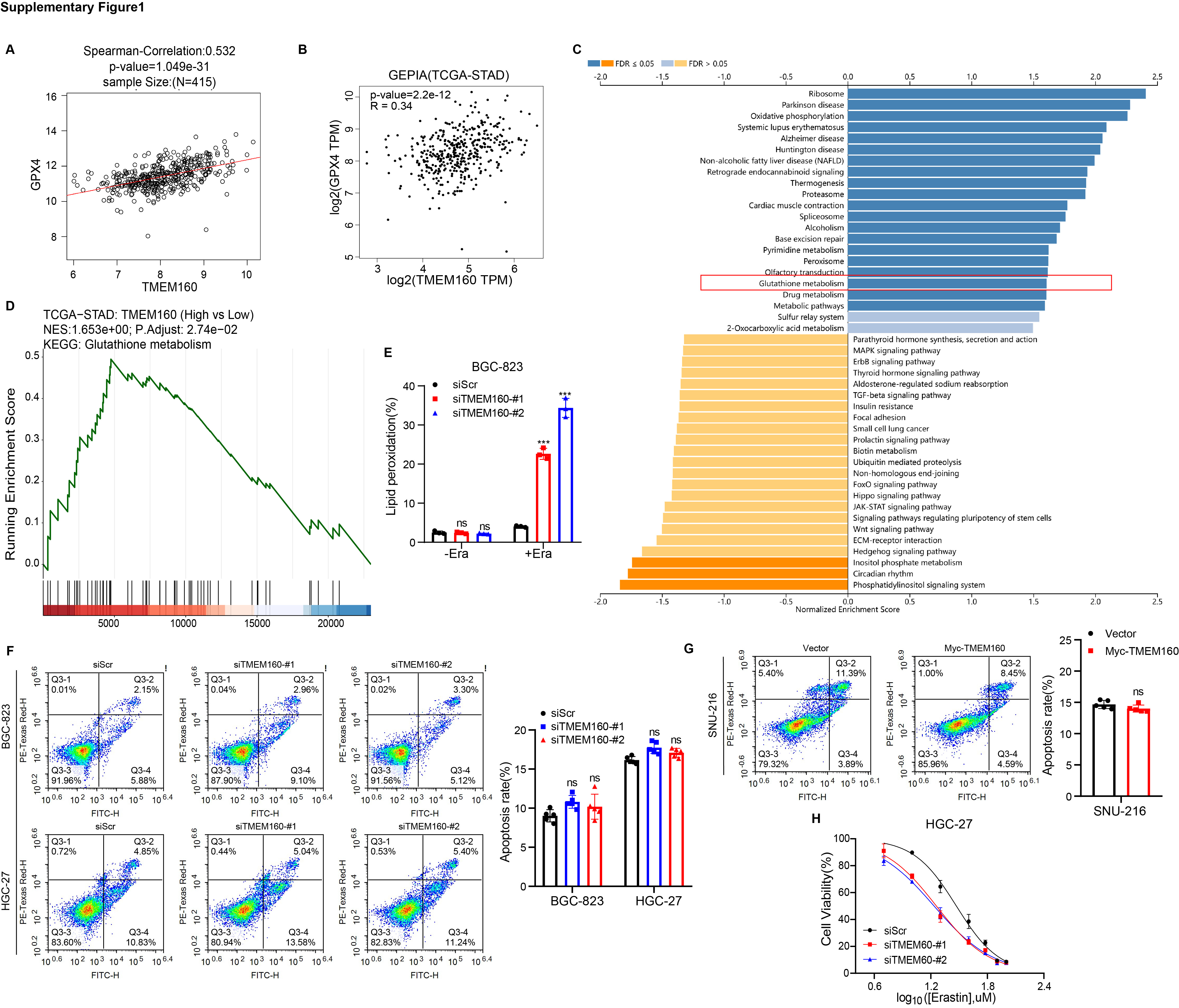
Supplementary Figures and Figure Legends**

**Supplementary Fig. 1 (A)**Correlation analysis between TMEM160 and GPX4 expression using the cBioPortal database; **(B)**Correlation analysis of TMEM160 and GPX4 mRNA levels using the GEPIA database; **(C)**KEGG analysis based on the LinkedOmics database showed that TMEM160 is associated with the glutathione metabolism pathway; **(D)**GSEA analysis based on the CAMOIP database showed a positive correlation between TMEM160 and glutathione metabolism; **(E)**Lipid peroxidation levels in BGC-823 cells transfected with siScr, siTMEM160-#1, and siTMEM160-#2 with or without Erastin treatment; **(F)**Apoptosis rates in BGC-823 and HGC-27 cells transfected with siScr, siTMEM160-#1, and siTMEM160-#2; **(G)**Apoptosis rates in SNU-216 cells transfected with vector and Myc-TMEM160 plasmids; **(H)**Therapeutic efficacy of Erastin on HGC-27 cells transfected with siScr, siTMEM160-#1, and siTMEM160-#2. Independent biological experiments were repeated at least three times, and the data are presented as the means ± SDs. Statistical differences are indicated by p-values,*p < 0.05, **p < **
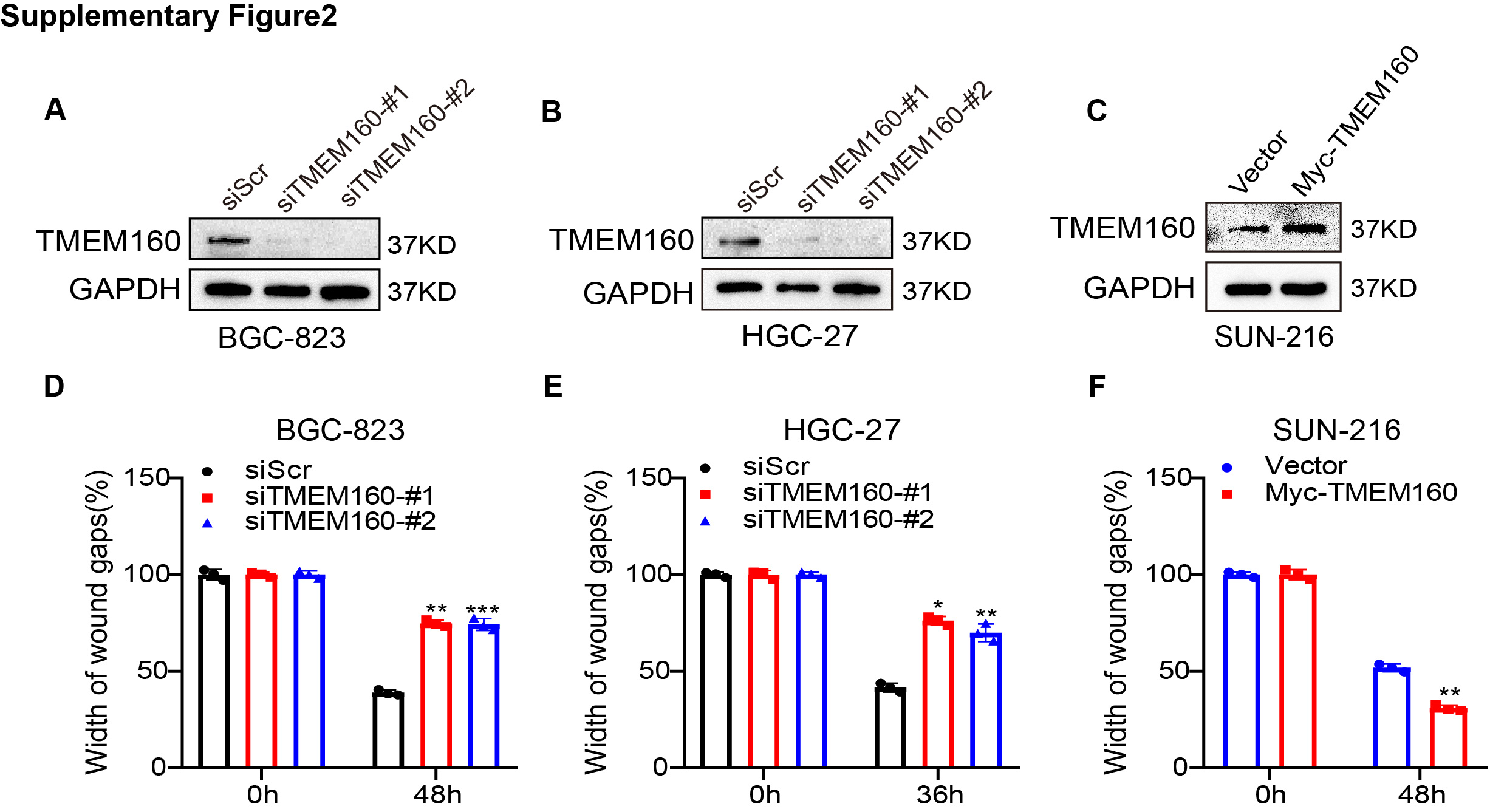
**0.01, and ***p < 0.001.

**Supplementary Fig. 2 (A and B)** TMEM160 protein levels in BGC-823 and HGC-27 cells transfected with siScr, siTMEM160-#1, and siTMEM160-#2;**(C)**TMEM160 protein levels in SNU-216 cells transfected with Vector and Myc-TMEM160; **(D and E)** Migration ability of BGC-823 and HGC-27 cells transfected with siScr, siTMEM160-#1, and siTMEM160-#2; **(F)**Migration ability of SNU-216 cells transfected with Vector and Myc-TMEM160. Independent biological experiments were repeated at least three times, and the data are presented as the means ± SDs. Statistical differences are indicated by p-values,*p < 0.05, **p < 0.01, and ***p < 0.001.

**
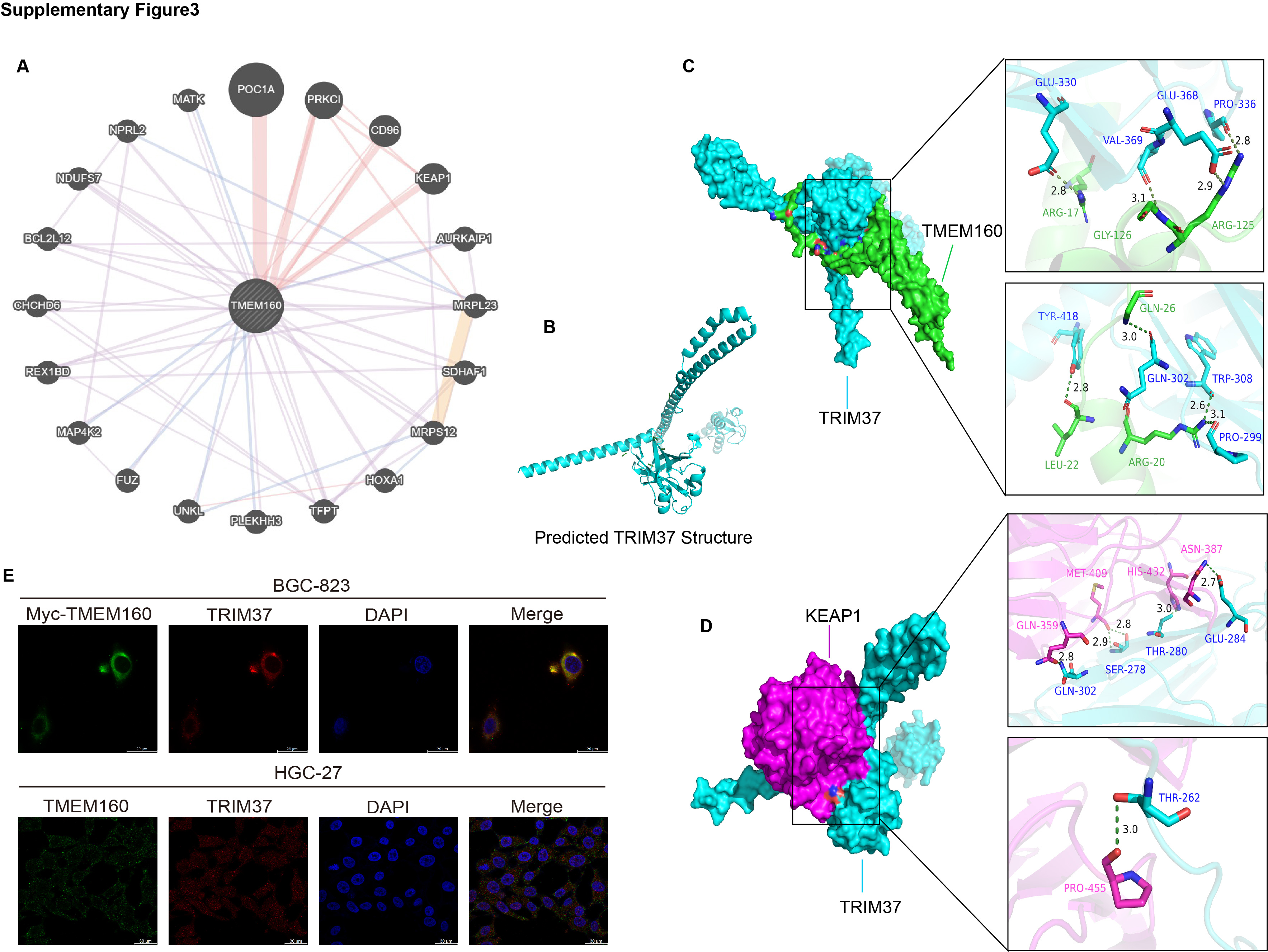
**

**Supplementary Fig. 3** **(A)**Identification of proteins interacting with TMEM160 using the GeneMANIA database; **(B)** Predicted 3D structural model of TRIM37 obtained from the UniProt database; **(C)**Predicted interface of one of the complexes formed by the binding of TMEM160 and TRIM37; **(D)**Predicted interface of one of the complexes formed by the binding of TRIM37 and KEAP1; **(E)**IF staining of TMEM160 and TRIM37 in BGC-823 and HGC-27 cells. Independent biological experiments were repeated at least three times.

**
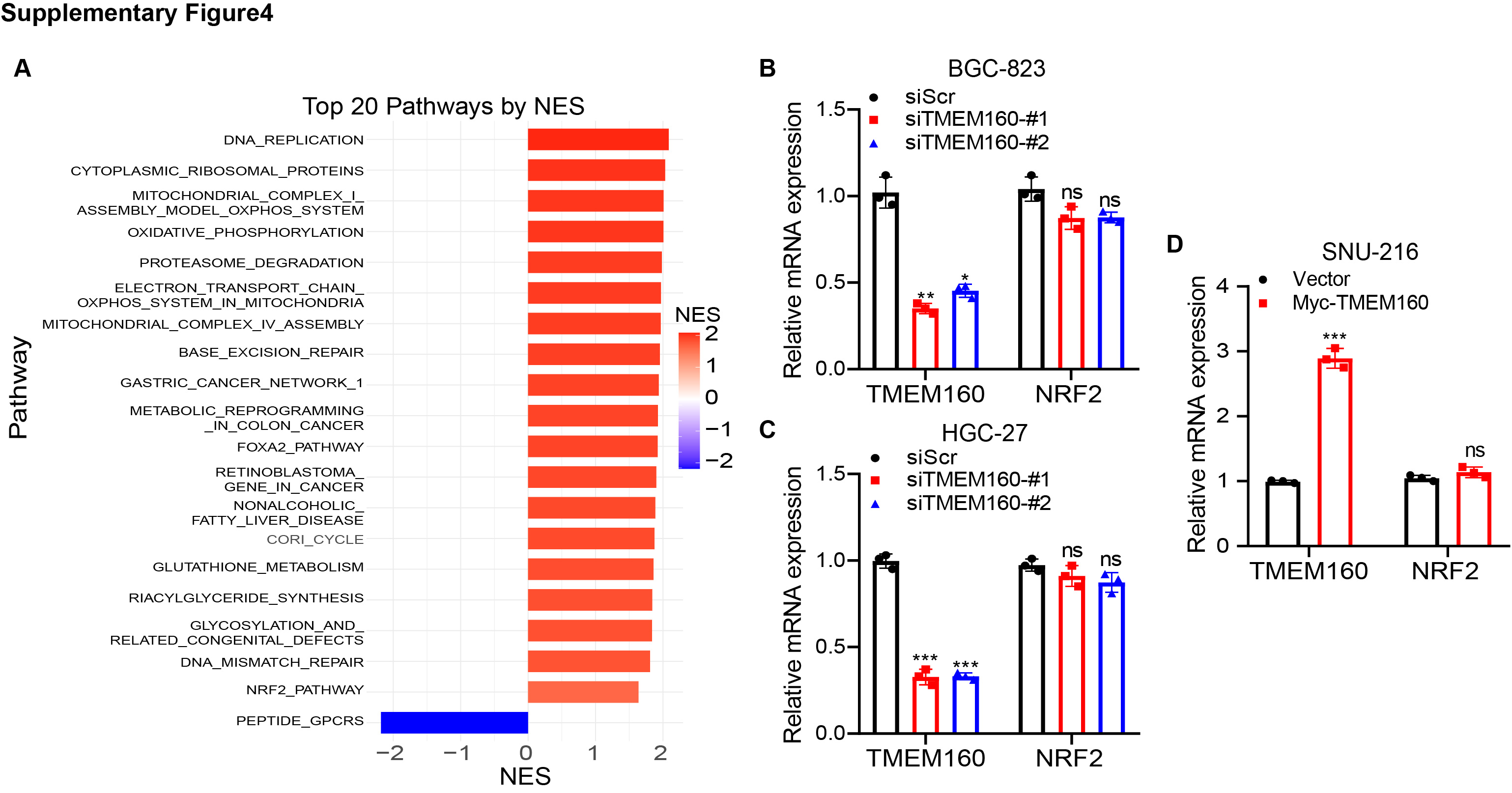
Supplementary Fig. 4 (A)**KEGG analysis based on the WikiPathways dataset of MSigDB database; **(B and C)** Downregulation of TMEM160 in BGC-823 and HGC-27 cells using siRNA, detection of NRF2 at the mRNA levels by RT-qPCR; **(D)**Upregulation of TMEM160 in SNU-216 cells using Myc-TMEM160 plasmids, detection of NRF2 at the mRNA level by RT-qPCR. Independent biological experiments were repeated at least three times, and the data are presented as the means ± SDs. Statistical differences are indicated by p-values of *p < 0.05, **p < 0.01, and ***p < 0.001.

**
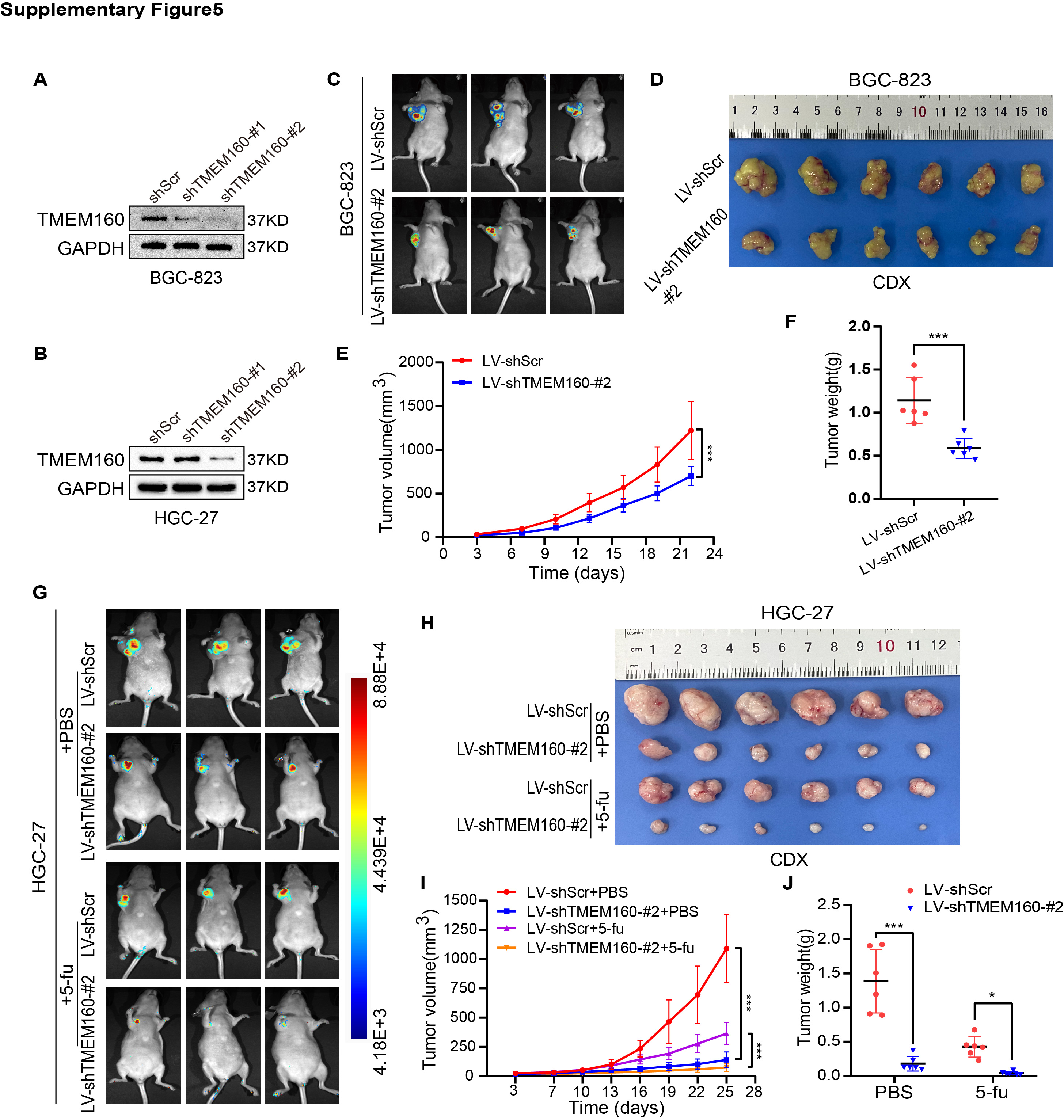
Supplementary Fig. 5 (A and B)** WB detection of TMEM160 protein expression in BGC-823 and HGC-27 cells infected with lentivirus encoding shRNA targeting TMEM160; BGC-823 cells stably transfected with the designated lentiviral vectors were subcutaneously implanted into female BALB/c nude mice to establish xenograft models, **(C)** Representative vivo images of xenograft tumors in the two indicated groups are shown; **(D)** Macroscopic images of tumors (n=6 per group); **(E)** Growth curves of tumor volume (n=6 per group); **(F)** Tumor weight (n=6 per group); HGC-27 cells stably transfected with the designated lentiviral vectors were subcutaneously implanted into female BALB/c nude mice to establish xenograft models, followed by the intraperitoneal injections of 5-fu and PBS. **(G)** Representative vivo images of xenograft tumors in the four indicated groups are shown.;**(H)** Macroscopic images of tumors (n=6 per group); **(I)** Growth curves of tumor volume (n=6 per group); **(J)** Tumor weight (n=6 per group). Independent biological experiments were repeated at least three times, and the data are presented as the means ± SDs. Statistical differences are indicated by p-values of *p < 0.05, **p < 0.01, and ***p < 0.001.

**
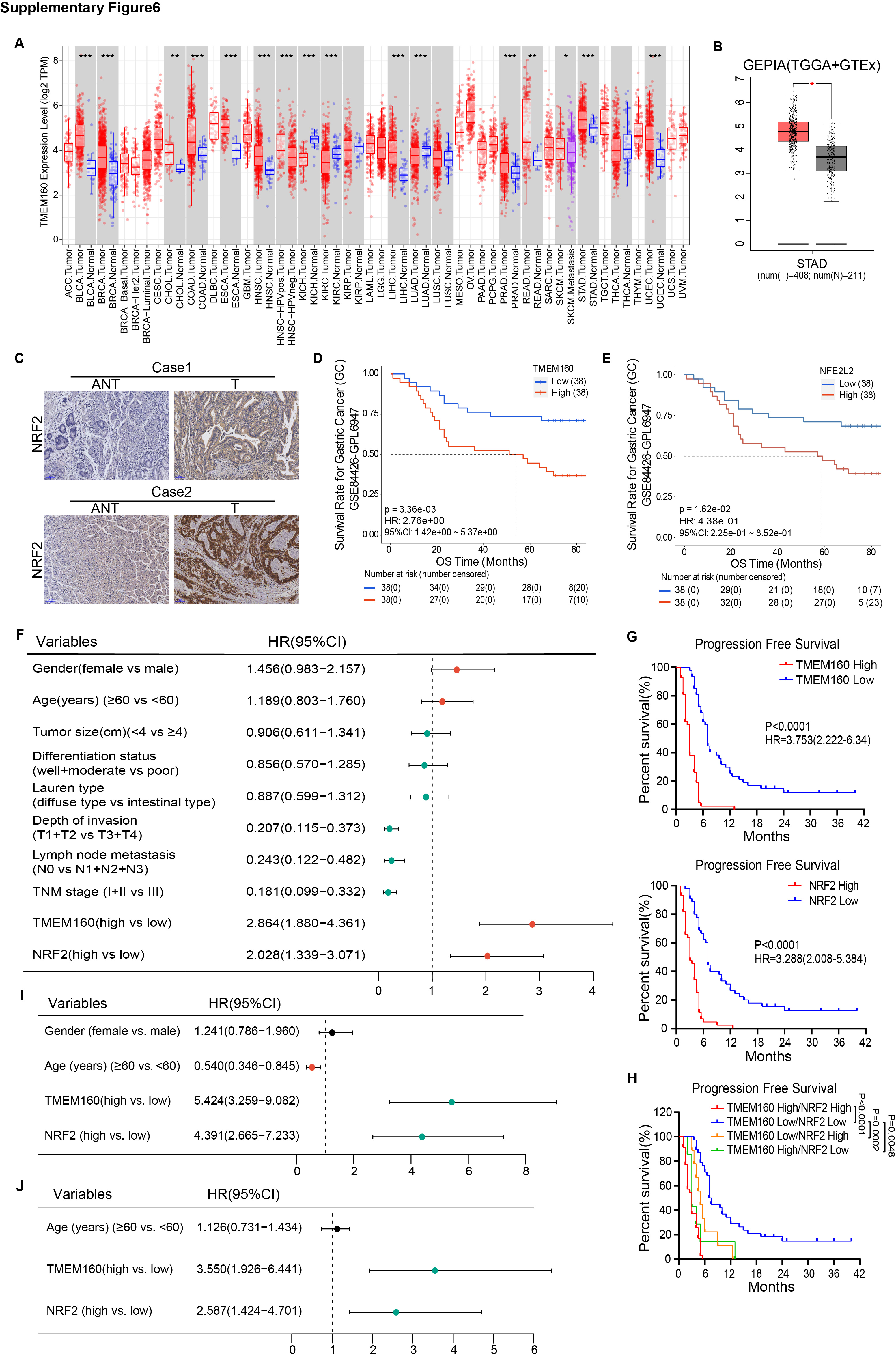
**

**Supplementary Fig. 6 (A)**Analysis of differential expression of TMEM160 in tumor and normal tissues across various cancers were based on the TIMER database; **(B)**Analysis of TMEM160 mRNA expression in gastric tumor tissues and normal tissues were based on the GEPIA database;**(C)** Representative IHC images of NRF2 expression of clinical gastric tumor tissue and adjacent normal tissue, scale bar: 100 μm; **(D and E)** Analysis of the relationship between TMEM160 or NRF2 expression and gastric cancer prognosis based on the TCGA STAD dataset of the PanCanSurvPlot database; **(F)**Univariate Cox regression analysis based on 180 GC patients who underwent surgical treatment; **(G-H)** Kaplan-Meier survival analysis based on 89 advanced GC patients; **(I and J)** Univariate and Multivariate Cox regression analysis based on 89 advanced GC patients. Statistical differences are indicated by p-values of *p < 0.05, **p < 0.01, and ***p < 0.001.

**Supplementary Tables**

**Supplementary Table 1. Sequences of siRNAs /LV-shRNAs in this research**

| **Name** | **Sequence** | |
| --- | --- | --- |
| siTMEM160-#1 | Sense:  Antisense: | 5’-CAUGCAGAGUGACAUGGGUTT-3’  5’-ACCCAUGUCACUCUGCAUGTT-3’ |
| siTMEM160-#2 | Sense:  Antisense: | 5’-CGAGGACUGGGACAUUAAATT-3’  5’-UUUAAUGUCCCAGUCCUCGTT-3’ |
| siNRF2-#1 | Sense:  Antisense: | 5’-CUGUUGAUUUAGACGGUAUTT-3’  5’-AUACCGUCUAAAUCAACAGTT-3’ |
| siNRF2-#2 | Sense:  Antisense: | 5’-CAGAAGUUGACAAUUAUCATT-3’  5’-UGAUAAUUGUCAACUUCUGTT-3’ |
| siNRF2-#3 | Sense:  Antisense: | 5’-CAGCUAUGGAGACACACUATT-3’  5’-UAGUGUGUCUCCAUAGCUGTT-3’ |
| siTRIM37-#1 | Sense:  Antisense: | 5’-GCUACGAGAACUAGUAAAUTT-3’  5’-AUUUACUAGUUCUCGUAGCTT-3’ |
| siTRIM37-#2 | Sense:  Antisense: | 5’-GCUGCACAGACUAGUUAUATT-3’  5’-UAUAACUAGUCUGUGCAGCTT-3’ |
| sh-TMEM160-#1 | - | 5’-ATGGGTCGGGAAGCAGCATAT-3’ |
| sh-TMEM160-#2 | - | 5’-TCATGCAGAGTGACATGGGTC-3’ |

**Supplementary Table 2. The primer sequences used in this research**

| **Gene** | **Sequence** |
| --- | --- |
| GAPDH-S | 5′-GGAAGCTTGTCATCAATGGAAATC-3′ |
| GAPDH-A | 5′-TGATGACCCTTTTGGCTCCC-3′ |
| TMEM160-S | 5′-TCCTCTCCTGGTTCCGCAA-3′ |
| TMEM160-A | 5′-CCCAGCAGGAAGAAGCCATAT-3′ |
| Keap1-S | 5′-CGCCAACTTCGCTGAGCA-3′ |
| Keap1-A | 5′-GAAGTTCGGCGTCAACGAGT-3′ |
| NRF2-S | 5′-GGATTTGATTGACATACTTTGGAGG-3′ |
| NRF2-A | 5′-TTTCTGACTGGATGTGCTGGG-3′ |
| SLC7A11-S | 5′- GGCAGTTGCTGGGCTGATTTA-3′ |
| SLC7A11-A | 5′-GATGACGAAGCCAATCCCTGT-3′ |
| GPX4-S | 5′-TGAAGATCCAACCCAAGGGC-3′ |
| GPX4-A | 5′-GACGGTGTCCAAACTTGGTG-3′ |

**Supplementary Table 3. Primary antibodies used in this research**

| **Primary antibody name** | **Source** | **ldentifier** |
| --- | --- | --- |
| TMEM160 | Abcam | AB185451 |
| GAPDH | Proteintech | 60004-1-Ig |
| KEAP1 | Proteintech | 10503-2-AP |
| KEAP1 | Origene | TA384599 |
| NRF2 | Proteintech | 16396-1-AP |
| GPX4 | Proteintech | 67763-1-Ig |
| SLC7A11 | Proteintech | 26864-1-AP |
| His-Tag | Proteintech | 66005-1-Ig |
| Flag-Tag | Proteintech | 66008–4-Ig |
| Flag-Tag | Proteintech | 20543-1-AP |
| HA-Tag | Proteintech | 51064–2-AP |
| HA-Tag | Proteintech | 66006-2-Ig |
| Myc-Tag | Proteintech | 60003-2-Ig |
| Myc-Tag | Proteintech | 16286-1-AP |
| Myc-Tag | Cell signaling technology | 71D10 |
| ubiquitin | Proteintech | 10201-2-AP |
| TRIM37 | Santa Cruz | sc-515044 |
| TRIM37 | Biomol GmbH | A301-174A |
| Rabbit IgG | Proteintech | B900610 |

**Supplementary Table 4. IC_50_ values of Erastin in BGC-823, HGC-27 and SNU-216 cells**

| **Group** | **Erastin（IC_50_ [ 95%CI ], uM）** | | |
| --- | --- | --- | --- |
|  | **BGC-823** | **HGC-27** | **SNU-216** |
| **siScr** | 59.61(56.30 to 63.35) | 29.58(27.72 to 31.53) | - |
| **siTMEM160-#1** | 26.01(23.98 to 28.14) | 18.15(16.97 to 19.40) | - |
| **siTMEM160-#2** | 28.70(26.70 to 30.79) | 17.15(16.32 to 18.02) | - |
| **Vector** | - | - | 193（166.8 to 230.2） |
| **Myc-TMEM160** | - | - | 462（405.0 to 541.2） |

**Supplementary Table 5. IC_50_ values of 5-fu and Oxaliplatin in BGC-823, HGC-27 and SNU-216 cells**

| **Group** | **5-fu（IC_50_[95%CI], uM）** | | | **Oxaliplatin（IC_50_[95%CI], uM）** | | |
| --- | --- | --- | --- | --- | --- | --- |
|  | **BGC-823** | **HGC-27** | **SNU-216** | **BGC-823** | **HGC-27** | **SNU-216** |
| **siScr** | 73.04(65.44 to 81.26) | 39.79(36.05 to 43.75) | - | 23.81（21.05 to 26.63） | 17.02(15.61 to 18.50) | - |
| **siTMEM160-#1** | 36.18(33.51 to 38.94) | 21.116(20.00 to22.29) | - | 10.29（9.665 to 10.96） | 8.40(7.764 to 9.071) | - |
| **siTMEM160-#2** | 37.54(34.72 to 40.45) | 18.67(17.94 to 19.42) | - | 11.49（10.66 to 12.38） | 8.803(8.30 to 9.33) | - |
| **Vector** | 74.7(69.46 to 80.27) | 42.31(38.33 to 46.72) | 245.4(229.4 to 262.5) | 23.73(21.05 to 26.62) | 16.93(15.8 to 18.13) | 42.43(34.15 to 53.83) |
| **Myc-TMEM160** | 163.9(142.8 to 187.2) | 86.5(80.3 to 93.28) | 933.7(857.1 to 1020) | 45.57(40.66 to 50.36) | 32.28(29.15 to 35.93) | 101.2(73.42 to 154.2) |

**Supplementary Table 6. IC_50_ values of 5-fu , Oxaliplatin and Erastin in BGC-823 and HGC-27 cells**

| **Group** | **5-fu（IC_50_ [95%CI], uM）** | | **Oxaliplatin（IC_50_ [95%CI], uM）** | | **Erastin（IC_50_ [95%CI], uM）** | |
| --- | --- | --- | --- | --- | --- | --- |
|  | **BGC-823** | **HGC-27** | **BGC-823** | **HGC-27** | **BGC-823** | **HGC-27** |
| **siScr**  **+Vector** | 73.79（63.33 to 84.91） | 43.57（41.22 to 45.99） | 25.44（23.47 to 27.41） | 16.54（14.72 to 18.45） | 55.47（51.60 to 59.67） | 28.34（27.35 to 29.35） |
| **Myc-TMEM160**  **+siScr** | 164.9（131.0 to 202.9） | 82.65（78.58 to 86.93） | 43.68（41.73 to 46.05） | 31.75（30.03 to 33.57） | 96.24（85.79 to 113.8） | 59.24（54.03 to 65.47） |
| **Myc-TMEM160**  **+siNRF2** | 63.31（59.07 to 67.63） | 38.23（35.56 to 40.98） | 22.23（20.49 to 23.97） | 16.17（14.50 to 17.93） | 43.65（41.45 to 45.94） | 23.09（22.09 to 24.13） |

**Supplementary Table 7. Proteins Interacting with TMEM160**

| **Interactor** | **Role** | **Organism** | **Throughput** | **HTP Score** |
| --- | --- | --- | --- | --- |
| [GABARAPL1](https://thebiogrid.org/117223/table/homo-sapiens/gabarapl1.html) | BAIT | H. sapiens | High | - |
| [GABARAPL2](https://thebiogrid.org/116473/table/homo-sapiens/gabarapl2.html) | BAIT | H. sapiens | High | - |
| [KBTBD7](https://thebiogrid.org/123873/table/homo-sapiens/kbtbd7.html) | BAIT | H. sapiens | High | - |
| [MAP1LC3A](https://thebiogrid.org/124137/table/homo-sapiens/map1lc3a.html) | BAIT | H. sapiens | High | - |
| [MAP1LC3B](https://thebiogrid.org/123565/table/homo-sapiens/map1lc3b.html) | BAIT | H. sapiens | High | - |
| [UBC](https://thebiogrid.org/113164/table/homo-sapiens/ubc.html) | BAIT | H. sapiens | High | - |
| [CEP135](https://thebiogrid.org/115018/table/homo-sapiens/cep135.html) | BAIT | H. sapiens | High | - |
| [MTMR4](https://thebiogrid.org/114560/table/homo-sapiens/mtmr4.html) | BAIT | H. sapiens | High | - |
| [RNF123](https://thebiogrid.org/121971/table/homo-sapiens/rnf123.html) | BAIT | H. sapiens | High | - |
| [EMP1](https://thebiogrid.org/108327/table/homo-sapiens/emp1.html) | HIT | H. sapiens | High | - |
| [SLC16A13](https://thebiogrid.org/128373/table/homo-sapiens/slc16a13.html) | HIT | H. sapiens | High | - |
| [PRKCI](https://thebiogrid.org/111570/table/homo-sapiens/prkci.html) | BAIT | H. sapiens | High | - |
| [KIAA1429](https://thebiogrid.org/117452/table/homo-sapiens/kiaa1429.html) | BAIT | H. sapiens | High | - |
| [SPOP](https://thebiogrid.org/113993/table/homo-sapiens/spop.html) | BAIT | H. sapiens | High | - |
| [DDRGK1](https://thebiogrid.org/122441/table/homo-sapiens/ddrgk1.html) | BAIT | H. sapiens | High | - |
| [NBR1](https://thebiogrid.org/110253/table/homo-sapiens/nbr1.html) | BAIT | H. sapiens | High | - |
| [SQSTM1](https://thebiogrid.org/114397/table/homo-sapiens/sqstm1.html) | BAIT | H. sapiens | High | - |
| [KEAP1](https://thebiogrid.org/115156/table/homo-sapiens/keap1.html) | BAIT | H. sapiens | High | 4.08 |
| [MFN2](https://thebiogrid.org/115255/table/homo-sapiens/mfn2.html) | BAIT | H. sapiens | High | 1 |
| [SQSTM1](https://thebiogrid.org/114397/table/homo-sapiens/sqstm1.html) | BAIT | H. sapiens | High | 0.9999 |
| [FAM19A5](https://thebiogrid.org/117345/table/homo-sapiens/fam19a5.html) | BAIT | H. sapiens | High | 0.9999 |
| [ASPH](https://thebiogrid.org/106936/table/homo-sapiens/asph.html) | BAIT | H. sapiens | High | 0.9995 |
| [VSIG1](https://thebiogrid.org/131072/table/homo-sapiens/vsig1.html) | BAIT | H. sapiens | High | 0.9993 |
| [HLA-DPB1](https://thebiogrid.org/109360/table/homo-sapiens/hla-dpb1.html) | BAIT | H. sapiens | High | 0.9991 |
| [S1PR1](https://thebiogrid.org/108225/table/homo-sapiens/s1pr1.html) | BAIT | H. sapiens | High | 0.999 |
| [ASPH](https://thebiogrid.org/106936/table/homo-sapiens/asph.html) | BAIT | H. sapiens | High | 0.9989 |
| [B3GAT3](https://thebiogrid.org/117620/table/homo-sapiens/b3gat3.html) | BAIT | H. sapiens | High | 0.9989 |
| [VSIG1](https://thebiogrid.org/131072/table/homo-sapiens/vsig1.html) | BAIT | H. sapiens | High | 0.9989 |
| [CD70](https://thebiogrid.org/107408/table/homo-sapiens/cd70.html) | BAIT | H. sapiens | High | 0.9988 |
| [LGALS3](https://thebiogrid.org/110149/table/homo-sapiens/lgals3.html) | BAIT | H. sapiens | High | 0.9986 |
| [P2RY2](https://thebiogrid.org/111068/table/homo-sapiens/p2ry2.html) | BAIT | H. sapiens | High | 0.9982 |
| [KBTBD7](https://thebiogrid.org/123873/table/homo-sapiens/kbtbd7.html) | BAIT | H. sapiens | High | 0.9979 |
| [MDFI](https://thebiogrid.org/110353/table/homo-sapiens/mdfi.html) | BAIT | H. sapiens | High | 0.9979 |
| [LPAR2](https://thebiogrid.org/114611/table/homo-sapiens/lpar2.html) | BAIT | H. sapiens | High | 0.9976 |
| [CAMKV](https://thebiogrid.org/122482/table/homo-sapiens/camkv.html) | BAIT | H. sapiens | High | 0.9967 |
| [PCDH9](https://thebiogrid.org/111134/table/homo-sapiens/pcdh9.html) | BAIT | H. sapiens | High | 0.9959 |
| [KBTBD7](https://thebiogrid.org/123873/table/homo-sapiens/kbtbd7.html) | BAIT | H. sapiens | High | 0.9958 |
| [CD44](https://thebiogrid.org/107398/table/homo-sapiens/cd44.html) | BAIT | H. sapiens | High | 0.9956 |
| [TXNDC11](https://thebiogrid.org/119253/table/homo-sapiens/txndc11.html) | BAIT | H. sapiens | High | 0.994 |
| [KLHL34](https://thebiogrid.org/129210/table/homo-sapiens/klhl34.html) | BAIT | H. sapiens | High | 0.9938 |
| [IL17B](https://thebiogrid.org/118065/table/homo-sapiens/il17b.html) | BAIT | H. sapiens | High | 0.9936 |
| [SCCPDH](https://thebiogrid.org/119286/table/homo-sapiens/sccpdh.html) | BAIT | H. sapiens | High | 0.9921 |
| [PLEKHM3](https://thebiogrid.org/132958/table/homo-sapiens/plekhm3.html) | BAIT | H. sapiens | High | 0.9916 |
| [PNPLA1](https://thebiogrid.org/130231/table/homo-sapiens/pnpla1.html) | BAIT | H. sapiens | High | 0.9914 |
| [S1PR4](https://thebiogrid.org/114243/table/homo-sapiens/s1pr4.html) | BAIT | H. sapiens | High | 0.991 |
| [TNFSF18](https://thebiogrid.org/114476/table/homo-sapiens/tnfsf18.html) | BAIT | H. sapiens | High | 0.9888 |
| [PRKCZ](https://thebiogrid.org/111576/table/homo-sapiens/prkcz.html) | BAIT | H. sapiens | High | 0.9879 |
| [BAGE2](https://thebiogrid.org/124473/table/homo-sapiens/bage2.html) | BAIT | H. sapiens | High | 0.986 |
| [SCCPDH](https://thebiogrid.org/119286/table/homo-sapiens/sccpdh.html) | BAIT | H. sapiens | High | 0.9791 |
| [C3AR1](https://thebiogrid.org/107180/table/homo-sapiens/c3ar1.html) | BAIT | H. sapiens | High | 0.9763 |
| [WDR90](https://thebiogrid.org/128250/table/homo-sapiens/wdr90.html) | BAIT | H. sapiens | High | 0.9733 |
| [AVPR2](https://thebiogrid.org/107035/table/homo-sapiens/avpr2.html) | BAIT | H. sapiens | High | 0.973 |
| [MIOX](https://thebiogrid.org/120733/table/homo-sapiens/miox.html) | BAIT | H. sapiens | High | 0.9712 |
| [CD44](https://thebiogrid.org/107398/table/homo-sapiens/cd44.html) | BAIT | H. sapiens | High | 0.9692 |
| [PIAS4](https://thebiogrid.org/119624/table/homo-sapiens/pias4.html) | BAIT | H. sapiens | High | 0.9623 |
| [SCCPDH](https://thebiogrid.org/119286/table/homo-sapiens/sccpdh.html) | BAIT | H. sapiens | High | 0.9503 |
| [FFAR1](https://thebiogrid.org/109122/table/homo-sapiens/ffar1.html) | BAIT | H. sapiens | High | 0.9494 |
| [IGSF8](https://thebiogrid.org/125011/table/homo-sapiens/igsf8.html) | BAIT | H. sapiens | High | 0.9482 |
| [PRKCI](https://thebiogrid.org/111570/table/homo-sapiens/prkci.html) | BAIT | H. sapiens | High | 0.9426 |
| [KCNMB2](https://thebiogrid.org/115536/table/homo-sapiens/kcnmb2.html) | BAIT | H. sapiens | High | 0.9387 |
| [GYPA](https://thebiogrid.org/109248/table/homo-sapiens/gypa.html) | BAIT | H. sapiens | High | 0.937 |
| [C3AR1](https://thebiogrid.org/107180/table/homo-sapiens/c3ar1.html) | BAIT | H. sapiens | High | 0.9365 |
| [CD44](https://thebiogrid.org/107398/table/homo-sapiens/cd44.html) | BAIT | H. sapiens | High | 0.9355 |
| [GPR156](https://thebiogrid.org/127920/table/homo-sapiens/gpr156.html) | BAIT | H. sapiens | High | 0.9213 |
| [C1ORF127](https://thebiogrid.org/127144/table/homo-sapiens/c1orf127.html) | BAIT | H. sapiens | High | 0.9166 |
| [CHRM5](https://thebiogrid.org/107555/table/homo-sapiens/chrm5.html) | BAIT | H. sapiens | High | 0.9159 |
| [VWCE](https://thebiogrid.org/128616/table/homo-sapiens/vwce.html) | BAIT | H. sapiens | High | 0.912 |
| [PTCH1](https://thebiogrid.org/111699/table/homo-sapiens/ptch1.html) | BAIT | H. sapiens | High | 0.9007 |
| [GLIPR1L2](https://thebiogrid.org/126842/table/homo-sapiens/glipr1l2.html) | BAIT | H. sapiens | High | 0.8947 |
| [CD96](https://thebiogrid.org/115519/table/homo-sapiens/cd96.html) | BAIT | H. sapiens | High | 0.8892 |
| [LRRC17](https://thebiogrid.org/115528/table/homo-sapiens/lrrc17.html) | BAIT | H. sapiens | High | 0.8855 |
| [CD274](https://thebiogrid.org/118891/table/homo-sapiens/cd274.html) | BAIT | H. sapiens | High | 0.8828 |
| [TSSK6](https://thebiogrid.org/123835/table/homo-sapiens/tssk6.html) | BAIT | H. sapiens | High | 0.8807 |
| [CHST8](https://thebiogrid.org/122148/table/homo-sapiens/chst8.html) | BAIT | H. sapiens | High | 0.8751 |
| [LY6G5C](https://thebiogrid.org/123285/table/homo-sapiens/ly6g5c.html) | BAIT | H. sapiens | High | 0.875 |
| [CD96](https://thebiogrid.org/115519/table/homo-sapiens/cd96.html) | BAIT | H. sapiens | High | 0.8672 |
| [C3AR1](https://thebiogrid.org/107180/table/homo-sapiens/c3ar1.html) | BAIT | H. sapiens | High | 0.8608 |
| [MOGS](https://thebiogrid.org/113599/table/homo-sapiens/mogs.html) | BAIT | H. sapiens | High | 0.8588 |
| [LRFN4](https://thebiogrid.org/122470/table/homo-sapiens/lrfn4.html) | BAIT | H. sapiens | High | 0.8553 |
| [MGAT4B](https://thebiogrid.org/116438/table/homo-sapiens/mgat4b.html) | BAIT | H. sapiens | High | 0.8544 |
| [NMUR1](https://thebiogrid.org/115600/table/homo-sapiens/nmur1.html) | BAIT | H. sapiens | High | 0.8533 |
| [VWCE](https://thebiogrid.org/128616/table/homo-sapiens/vwce.html) | BAIT | H. sapiens | High | 0.8226 |
| [AVPR2](https://thebiogrid.org/107035/table/homo-sapiens/avpr2.html) | BAIT | H. sapiens | High | 0.8178 |
| [SPAG11A](https://thebiogrid.org/575769/table/homo-sapiens/spag11a.html) | BAIT | H. sapiens | High | 0.812 |
| [FZD10](https://thebiogrid.org/116380/table/homo-sapiens/fzd10.html) | BAIT | H. sapiens | High | 0.8074 |
| [DCAF10](https://thebiogrid.org/122612/table/homo-sapiens/dcaf10.html) | BAIT | H. sapiens | High | 0.7978 |
| [CHST8](https://thebiogrid.org/122148/table/homo-sapiens/chst8.html) | BAIT | H. sapiens | High | 0.7935 |
| [CD274](https://thebiogrid.org/118891/table/homo-sapiens/cd274.html) | BAIT | H. sapiens | High | 0.7919 |
| [ARHGEF16](https://thebiogrid.org/118085/table/homo-sapiens/arhgef16.html) | BAIT | H. sapiens | High | 0.7916 |
| [CD96](https://thebiogrid.org/115519/table/homo-sapiens/cd96.html) | BAIT | H. sapiens | High | 0.7901 |
| [REEP5](https://thebiogrid.org/113637/table/homo-sapiens/reep5.html) | BAIT | H. sapiens | High | 0.7869 |
| [ADCK5](https://thebiogrid.org/128442/table/homo-sapiens/adck5.html) | BAIT | H. sapiens | High | 0.7832 |
| [GADD45GIP1](https://thebiogrid.org/124721/table/homo-sapiens/gadd45gip1.html) | BAIT | H. sapiens | High | 0.7756 |
| [PTCH1](https://thebiogrid.org/111699/table/homo-sapiens/ptch1.html) | BAIT | H. sapiens | High | 0.7724 |
| [CUL9](https://thebiogrid.org/116736/table/homo-sapiens/cul9.html) | BAIT | H. sapiens | High | 0.7622 |
| [PNPLA1](https://thebiogrid.org/130231/table/homo-sapiens/pnpla1.html) | BAIT | H. sapiens | High | 0.7594 |
| [IGSF8](https://thebiogrid.org/125011/table/homo-sapiens/igsf8.html) | BAIT | H. sapiens | High | 0.7574 |
| [UCN3](https://thebiogrid.org/125284/table/homo-sapiens/ucn3.html) | BAIT | H. sapiens | High | 0.7255 |
| [CD3E](https://thebiogrid.org/107354/table/homo-sapiens/cd3e.html) | BAIT | H. sapiens | High | 0.7215 |
| [C1ORF61](https://thebiogrid.org/115748/table/homo-sapiens/c1orf61.html) | BAIT | H. sapiens | High | 0.7213 |
| [FAM150B](https://thebiogrid.org/129997/table/homo-sapiens/fam150b.html) | BAIT | H. sapiens | High | 0.6876 |
| [SPANXN2](https://thebiogrid.org/138938/table/homo-sapiens/spanxn2.html) | BAIT | H. sapiens | High | 0.6876 |
| [CCIN](https://thebiogrid.org/107324/table/homo-sapiens/ccin.html) | BAIT | H. sapiens | High | 0.6701 |
| [TINAG](https://thebiogrid.org/118107/table/homo-sapiens/tinag.html) | BAIT | H. sapiens | High | 0.6627 |
| [ASGR1](https://thebiogrid.org/106924/table/homo-sapiens/asgr1.html) | BAIT | H. sapiens | High | 0.6341 |
| [DRD2](https://thebiogrid.org/108147/table/homo-sapiens/drd2.html) | BAIT | H. sapiens | High | 0.6335 |
| [EMID1](https://thebiogrid.org/126183/table/homo-sapiens/emid1.html) | BAIT | H. sapiens | High | 0.5968 |
| [PLCG2](https://thebiogrid.org/111352/table/homo-sapiens/plcg2.html) | BAIT | H. sapiens | High | 0.4764 |
| [CBLB](https://thebiogrid.org/107316/table/homo-sapiens/cblb.html) | BAIT | H. sapiens | High | 0.4764 |
| [SCCPDH](https://thebiogrid.org/119286/table/homo-sapiens/sccpdh.html) | BAIT | H. sapiens | High | 0.4526 |
| [TRAF1](https://thebiogrid.org/113037/table/homo-sapiens/traf1.html) | BAIT | H. sapiens | High | 0.4526 |
| [IQCF2](https://thebiogrid.org/132986/table/homo-sapiens/iqcf2.html) | BAIT | H. sapiens | High | 0.4475 |
| [UPP1](https://thebiogrid.org/113224/table/homo-sapiens/upp1.html) | BAIT | H. sapiens | High | 0.4475 |
| [MRPL53](https://thebiogrid.org/125521/table/homo-sapiens/mrpl53.html) | BAIT | H. sapiens | High | 0.4048 |

**Supplementary Table 8. Proteins Interacting with KEAP1**

| **Interactor** | **Role** | **Organism** | **Throughput** | **HTP Score** |
| --- | --- | --- | --- | --- |
| [ACD](https://thebiogrid.org/122379/table/homo-sapiens/acd.html) | BAIT | H. sapiens | High | 0.7991 |
| [AK4](https://thebiogrid.org/106708/table/homo-sapiens/ak4.html) | BAIT | H. sapiens | High | - |
| [AMBRA1](https://thebiogrid.org/120765/table/homo-sapiens/ambra1.html) | BAIT | H. sapiens | High | - |
| [ANKS1A](https://thebiogrid.org/116889/table/homo-sapiens/anks1a.html) | BAIT | H. sapiens | High | - |
| [ANLN](https://thebiogrid.org/119959/table/homo-sapiens/anln.html) | BAIT | H. sapiens | High | - |
| [APP](https://thebiogrid.org/106848/table/homo-sapiens/app.html) | BAIT | H. sapiens | High | 0.9398 |
| [ARCN1](https://thebiogrid.org/106867/table/homo-sapiens/arcn1.html) | HIT | H. sapiens | High | 2.36 |
| [ASTN1](https://thebiogrid.org/106952/table/homo-sapiens/astn1.html) | HIT | H. sapiens | High | - |
| [ATG16L1](https://thebiogrid.org/120375/table/homo-sapiens/atg16l1.html) | BAIT | H. sapiens | High | - |
| [ATG5](https://thebiogrid.org/114859/table/homo-sapiens/atg5.html) | BAIT | H. sapiens | High | - |
| [ATP6V0A2](https://thebiogrid.org/117089/table/homo-sapiens/atp6v0a2.html) | HIT | H. sapiens | High | - |
| [BAD](https://thebiogrid.org/107048/table/homo-sapiens/bad.html) | BAIT | H. sapiens | High | - |
| [BAG3](https://thebiogrid.org/114907/table/homo-sapiens/bag3.html) | BAIT | H. sapiens | High | - |
| [BBS1](https://thebiogrid.org/107058/table/homo-sapiens/bbs1.html) | HIT | H. sapiens | High | 13.06 |
| [BCL11B](https://thebiogrid.org/122343/table/homo-sapiens/bcl11b.html) | BAIT | H. sapiens | High | 0.8583 |
| [BOD1L1](https://thebiogrid.org/129238/table/homo-sapiens/bod1l1.html) | BAIT | H. sapiens | High | - |
| [BPTF](https://thebiogrid.org/108481/table/homo-sapiens/bptf.html) | HIT | H. sapiens | High | - |
| [BRCA2](https://thebiogrid.org/107142/table/homo-sapiens/brca2.html) | HIT | H. sapiens | High | - |
| [BRD7](https://thebiogrid.org/118883/table/homo-sapiens/brd7.html) | BAIT | H. sapiens | High | - |
| [BRPF1](https://thebiogrid.org/113614/table/homo-sapiens/brpf1.html) | HIT | H. sapiens | High | - |
| [BTRC](https://thebiogrid.org/114457/table/homo-sapiens/btrc.html) | BAIT | H. sapiens | High | - |
| [C2ORF68](https://thebiogrid.org/132926/table/homo-sapiens/c2orf68.html) | HIT | H. sapiens | High | - |
| [C6ORF132](https://thebiogrid.org/571814/table/homo-sapiens/c6orf132.html) | HIT | H. sapiens | High | - |
| [CALCOCO2](https://thebiogrid.org/115535/table/homo-sapiens/calcoco2.html) | BAIT | H. sapiens | High | - |
| [CANT1](https://thebiogrid.org/125875/table/homo-sapiens/cant1.html) | HIT | H. sapiens | High | - |
| [CARD10](https://thebiogrid.org/118908/table/homo-sapiens/card10.html) | HIT | H. sapiens | High | - |
| [CAT](https://thebiogrid.org/107297/table/homo-sapiens/cat.html) | BAIT | H. sapiens | High | - |
| [CAV1](https://thebiogrid.org/107305/table/homo-sapiens/cav1.html) | BAIT | H. sapiens | High | 4.95 |
| [CCDC71L](https://thebiogrid.org/127965/table/homo-sapiens/ccdc71l.html) | HIT | H. sapiens | High | - |
| [CCNB1](https://thebiogrid.org/107332/table/homo-sapiens/ccnb1.html) | BAIT | H. sapiens | High | - |
| [CCNB1IP1](https://thebiogrid.org/121779/table/homo-sapiens/ccnb1ip1.html) | HIT | H. sapiens | High | - |
| [CCND1](https://thebiogrid.org/107067/table/homo-sapiens/ccnd1.html) | BAIT | H. sapiens | High | - |
| [CCNF](https://thebiogrid.org/107339/table/homo-sapiens/ccnf.html) | BAIT | H. sapiens | High | - |
| [CDC34](https://thebiogrid.org/107432/table/homo-sapiens/cdc34.html) | BAIT | H. sapiens | High | - |
| [CDH1](https://thebiogrid.org/107434/table/homo-sapiens/cdh1.html) | BAIT | H. sapiens | High | - |
| [CDH5](https://thebiogrid.org/107438/table/homo-sapiens/cdh5.html) | BAIT | H. sapiens | High | 0.9933 |
| [CDK20](https://thebiogrid.org/117096/table/homo-sapiens/cdk20.html) | BAIT | H. sapiens | High | 0.4996 |
| [CDK6](https://thebiogrid.org/107456/table/homo-sapiens/cdk6.html) | BAIT | H. sapiens | High | - |
| [CEP135](https://thebiogrid.org/115018/table/homo-sapiens/cep135.html) | BAIT | H. sapiens | High | - |
| [CIAO1](https://thebiogrid.org/114791/table/homo-sapiens/ciao1.html) | BAIT | H. sapiens | High | - |
| [CLU](https://thebiogrid.org/107603/table/homo-sapiens/clu.html) | BAIT | H. sapiens | High | - |
| [COA3](https://thebiogrid.org/118785/table/homo-sapiens/coa3.html) | HIT | H. sapiens | High | - |
| [COL1A1](https://thebiogrid.org/107674/table/homo-sapiens/col1a1.html) | HIT | H. sapiens | High | - |
| [COL2A1](https://thebiogrid.org/107677/table/homo-sapiens/col2a1.html) | HIT | H. sapiens | High | - |
| [COPG1](https://thebiogrid.org/116496/table/homo-sapiens/copg1.html) | HIT | H. sapiens | High | 2.04 |
| [COQ2](https://thebiogrid.org/118083/table/homo-sapiens/coq2.html) | HIT | H. sapiens | High | - |
| [CSNK2A1](https://thebiogrid.org/107841/table/homo-sapiens/csnk2a1.html) | HIT | H. sapiens | High | - |
| [CSTF2T](https://thebiogrid.org/116881/table/homo-sapiens/cstf2t.html) | HIT | H. sapiens | High | 0.4794 |
| [CSTF3](https://thebiogrid.org/107861/table/homo-sapiens/cstf3.html) | HIT | H. sapiens | High | 0.4253 |
| [CTBP2](https://thebiogrid.org/107870/table/homo-sapiens/ctbp2.html) | HIT | H. sapiens | High | - |
| [CUL3](https://thebiogrid.org/114030/table/homo-sapiens/cul3.html) | BAIT | H. sapiens | High | - |
| [CUL4A](https://thebiogrid.org/114029/table/homo-sapiens/cul4a.html) | BAIT | H. sapiens | High | - |
| [DCAF10](https://thebiogrid.org/122612/table/homo-sapiens/dcaf10.html) | BAIT | H. sapiens | High | - |
| [DCAF11](https://thebiogrid.org/123251/table/homo-sapiens/dcaf11.html) | BAIT | H. sapiens | High | - |
| [DCAF4](https://thebiogrid.org/117545/table/homo-sapiens/dcaf4.html) | BAIT | H. sapiens | High | - |
| [DCAF6](https://thebiogrid.org/120933/table/homo-sapiens/dcaf6.html) | BAIT | H. sapiens | High | - |
| [DDX58](https://thebiogrid.org/117121/table/homo-sapiens/ddx58.html) | BAIT | H. sapiens | High | - |
| [DEPTOR](https://thebiogrid.org/122304/table/homo-sapiens/deptor.html) | BAIT | H. sapiens | High | - |
| [DFNB31](https://thebiogrid.org/117381/table/homo-sapiens/dfnb31.html) | HIT | H. sapiens | High | - |
| [DNAJB8](https://thebiogrid.org/127919/table/homo-sapiens/dnajb8.html) | BAIT | H. sapiens | High | 0.8434 |
| [DPF1](https://thebiogrid.org/113836/table/homo-sapiens/dpf1.html) | BAIT | H. sapiens | High | 0.8201 |
| [DPP9](https://thebiogrid.org/124789/table/homo-sapiens/dpp9.html) | BAIT | H. sapiens | High | 0.9747 |
| [DYRK1A](https://thebiogrid.org/108192/table/homo-sapiens/dyrk1a.html) | HIT | H. sapiens | High | - |
| [EAF1](https://thebiogrid.org/124514/table/homo-sapiens/eaf1.html) | HIT | H. sapiens | High | - |
| [EEA1](https://thebiogrid.org/113999/table/homo-sapiens/eea1.html) | BAIT | H. sapiens | High | - |
| [EEF2](https://thebiogrid.org/108258/table/homo-sapiens/eef2.html) | HIT | H. sapiens | High | - |
| [EGFR](https://thebiogrid.org/108276/table/homo-sapiens/egfr.html) | BAIT | H. sapiens | High | - |
| [EGLN3](https://thebiogrid.org/125185/table/homo-sapiens/egln3.html) | BAIT | H. sapiens | High | - |
| [ELMO2](https://thebiogrid.org/121987/table/homo-sapiens/elmo2.html) | HIT | H. sapiens | High | - |
| [ERICH5](https://thebiogrid.org/128453/table/homo-sapiens/erich5.html) | HIT | H. sapiens | High | - |
| [ERN2](https://thebiogrid.org/115843/table/homo-sapiens/ern2.html) | BAIT | H. sapiens | High | 1088 |
| [ESR2](https://thebiogrid.org/108404/table/homo-sapiens/esr2.html) | BAIT | H. sapiens | High | - |
| [ETF1](https://thebiogrid.org/108408/table/homo-sapiens/etf1.html) | HIT | H. sapiens | High | 5 |
| [EZH1](https://thebiogrid.org/108445/table/homo-sapiens/ezh1.html) | HIT | H. sapiens | High | - |
| [FAM129B](https://thebiogrid.org/122328/table/homo-sapiens/fam129b.html) | HIT | H. sapiens | High | 9.99 |
| [FAM133B](https://thebiogrid.org/129218/table/homo-sapiens/fam133b.html) | HIT | H. sapiens | High | 0.9249 |
| [FAM20C](https://thebiogrid.org/121294/table/homo-sapiens/fam20c.html) | BAIT | H. sapiens | High | - |
| [FAM3A](https://thebiogrid.org/121898/table/homo-sapiens/fam3a.html) | HIT | H. sapiens | High | 1 |
| [FAM46A](https://thebiogrid.org/120744/table/homo-sapiens/fam46a.html) | BAIT | H. sapiens | High | - |
| [FAM69A](https://thebiogrid.org/132793/table/homo-sapiens/fam69a.html) | HIT | H. sapiens | High | - |
| [FASN](https://thebiogrid.org/108488/table/homo-sapiens/fasn.html) | BAIT | H. sapiens | High | - |
| [FBXW11](https://thebiogrid.org/116887/table/homo-sapiens/fbxw11.html) | BAIT | H. sapiens | High | - |
| [FBXW8](https://thebiogrid.org/117645/table/homo-sapiens/fbxw8.html) | BAIT | H. sapiens | High | - |
| [FERMT3](https://thebiogrid.org/123735/table/homo-sapiens/fermt3.html) | BAIT | H. sapiens | High | - |
| [FKRP](https://thebiogrid.org/122565/table/homo-sapiens/fkrp.html) | HIT | H. sapiens | High | - |
| [G6PC](https://thebiogrid.org/108813/table/homo-sapiens/g6pc.html) | HIT | H. sapiens | High | - |
| [GABARAP](https://thebiogrid.org/116465/table/homo-sapiens/gabarap.html) | BAIT | H. sapiens | High | - |
| [GABARAPL1](https://thebiogrid.org/117223/table/homo-sapiens/gabarapl1.html) | BAIT | H. sapiens | High | - |
| [GABARAPL2](https://thebiogrid.org/116473/table/homo-sapiens/gabarapl2.html) | BAIT | H. sapiens | High | - |
| [GAR1](https://thebiogrid.org/119949/table/homo-sapiens/gar1.html) | BAIT | H. sapiens | High | - |
| [GBAS](https://thebiogrid.org/108901/table/homo-sapiens/gbas.html) | HIT | H. sapiens | High | 4.56 |
| [GET4](https://thebiogrid.org/119636/table/homo-sapiens/get4.html) | HIT | H. sapiens | High | 2.36 |
| [GLRX5](https://thebiogrid.org/119386/table/homo-sapiens/glrx5.html) | BAIT | H. sapiens | High | - |
| [GNB2](https://thebiogrid.org/109045/table/homo-sapiens/gnb2.html) | BAIT | H. sapiens | High | - |
| [GPR151](https://thebiogrid.org/126397/table/homo-sapiens/gpr151.html) | HIT | H. sapiens | High | - |
| [GPRASP2](https://thebiogrid.org/125399/table/homo-sapiens/gprasp2.html) | BAIT | H. sapiens | High | - |
| [GPRC5A](https://thebiogrid.org/114514/table/homo-sapiens/gprc5a.html) | HIT | H. sapiens | High | - |
| [GRIN3B](https://thebiogrid.org/125509/table/homo-sapiens/grin3b.html) | HIT | H. sapiens | High | - |
| [GSK3A](https://thebiogrid.org/109186/table/homo-sapiens/gsk3a.html) | BAIT | H. sapiens | High | - |
| [GSK3B](https://thebiogrid.org/109187/table/homo-sapiens/gsk3b.html) | BAIT | H. sapiens | High | - |
| [GSPT1](https://thebiogrid.org/109190/table/homo-sapiens/gspt1.html) | BAIT | H. sapiens | High | - |
| [GTPBP6](https://thebiogrid.org/113858/table/homo-sapiens/gtpbp6.html) | HIT | H. sapiens | High | 1 |
| [GYPA](https://thebiogrid.org/109248/table/homo-sapiens/gypa.html) | BAIT | H. sapiens | High | - |
| [HDAC1](https://thebiogrid.org/109315/table/homo-sapiens/hdac1.html) | BAIT | H. sapiens | High | - |
| [HDGFRP2](https://thebiogrid.org/124221/table/homo-sapiens/hdgfrp2.html) | HIT | H. sapiens | High | 0.9687 |
| [HSD11B1L](https://thebiogrid.org/131927/table/homo-sapiens/hsd11b1l.html) | HIT | H. sapiens | High | - |
| [HSPA8](https://thebiogrid.org/109544/table/homo-sapiens/hspa8.html) | BAIT | H. sapiens | High | - |
| [ICAM1](https://thebiogrid.org/109610/table/homo-sapiens/icam1.html) | HIT | H. sapiens | High | - |
| [IFIH1](https://thebiogrid.org/122082/table/homo-sapiens/ifih1.html) | HIT | H. sapiens | High | - |
| [IL17F](https://thebiogrid.org/125201/table/homo-sapiens/il17f.html) | BAIT | H. sapiens | High | 0.8059 |
| [IQCC](https://thebiogrid.org/120843/table/homo-sapiens/iqcc.html) | HIT | H. sapiens | High | - |
| [IRF1](https://thebiogrid.org/109867/table/homo-sapiens/irf1.html) | BAIT | H. sapiens | High | - |
| [KDM1A](https://thebiogrid.org/116667/table/homo-sapiens/kdm1a.html) | BAIT | H. sapiens | High | - |
| [KEAP1](https://thebiogrid.org/115156/table/homo-sapiens/keap1.html) | HIT | H. sapiens | High | - |
| [KIF14](https://thebiogrid.org/115256/table/homo-sapiens/kif14.html) | BAIT | H. sapiens | High | - |
| [KLHL1](https://thebiogrid.org/121671/table/homo-sapiens/klhl1.html) | HIT | H. sapiens | High | 0.0388 |
| [KLHL12](https://thebiogrid.org/121890/table/homo-sapiens/klhl12.html) | HIT | H. sapiens | High | 0.0103 |
| [KLHL17](https://thebiogrid.org/130885/table/homo-sapiens/klhl17.html) | HIT | H. sapiens | High | 0.0005 |
| [KLHL2](https://thebiogrid.org/116431/table/homo-sapiens/klhl2.html) | BAIT | H. sapiens | High | 0.9841 |
| [KLHL28](https://thebiogrid.org/120170/table/homo-sapiens/klhl28.html) | HIT | H. sapiens | High | 0.0031 |
| [KLHL3](https://thebiogrid.org/117637/table/homo-sapiens/klhl3.html) | BAIT | H. sapiens | High | 0.9536 |
| [KLHL5](https://thebiogrid.org/119278/table/homo-sapiens/klhl5.html) | HIT | H. sapiens | High | 0.0029 |
| [LAMA1](https://thebiogrid.org/129792/table/homo-sapiens/lama1.html) | HIT | H. sapiens | High | - |
| [LAMC1](https://thebiogrid.org/110109/table/homo-sapiens/lamc1.html) | HIT | H. sapiens | High | - |
| [LAMTOR5](https://thebiogrid.org/115796/table/homo-sapiens/lamtor5.html) | BAIT | H. sapiens | High | - |
| [LDLR](https://thebiogrid.org/110141/table/homo-sapiens/ldlr.html) | BAIT | H. sapiens | High | - |
| [LEPREL2](https://thebiogrid.org/115790/table/homo-sapiens/leprel2.html) | BAIT | H. sapiens | High | 0.9812 |
| [LSM3](https://thebiogrid.org/118105/table/homo-sapiens/lsm3.html) | HIT | H. sapiens | High | - |
| [MAD2L1](https://thebiogrid.org/110260/table/homo-sapiens/mad2l1.html) | HIT | H. sapiens | High | 1.44 |
| [MAP1LC3A](https://thebiogrid.org/124137/table/homo-sapiens/map1lc3a.html) | BAIT | H. sapiens | High | - |
| [MAPK14](https://thebiogrid.org/107819/table/homo-sapiens/mapk14.html) | HIT | H. sapiens | High | - |
| [MAPK8IP1](https://thebiogrid.org/114864/table/homo-sapiens/mapk8ip1.html) | BAIT | H. sapiens | High | 0.9616 |
| [MAPKBP1](https://thebiogrid.org/116650/table/homo-sapiens/mapkbp1.html) | HIT | H. sapiens | High | - |
| [MAZ](https://thebiogrid.org/110320/table/homo-sapiens/maz.html) | HIT | H. sapiens | High | 2.04 |
| [MCAM](https://thebiogrid.org/110332/table/homo-sapiens/mcam.html) | BAIT | H. sapiens | High | - |
| [MCC](https://thebiogrid.org/110333/table/homo-sapiens/mcc.html) | HIT | H. sapiens | High | - |
| [MCM3](https://thebiogrid.org/110340/table/homo-sapiens/mcm3.html) | BAIT | H. sapiens | High | - |
| [MCMBP](https://thebiogrid.org/122976/table/homo-sapiens/mcmbp.html) | HIT | H. sapiens | High | 6.38 |
| [MDFI](https://thebiogrid.org/110353/table/homo-sapiens/mdfi.html) | BAIT | H. sapiens | High | 0.7288 |
| [MED13](https://thebiogrid.org/115294/table/homo-sapiens/med13.html) | HIT | H. sapiens | High | - |
| [MED4](https://thebiogrid.org/118849/table/homo-sapiens/med4.html) | BAIT | H. sapiens | High | - |
| [MED9](https://thebiogrid.org/120403/table/homo-sapiens/med9.html) | HIT | H. sapiens | High | - |
| [MEF2BNB](https://thebiogrid.org/610381/table/homo-sapiens/mef2bnb.html) | HIT | H. sapiens | High | - |
| [MLST8](https://thebiogrid.org/122113/table/homo-sapiens/mlst8.html) | BAIT | H. sapiens | High | 0.8703 |
| [MRRF](https://thebiogrid.org/124944/table/homo-sapiens/mrrf.html) | HIT | H. sapiens | High | - |
| [MYH7](https://thebiogrid.org/110710/table/homo-sapiens/myh7.html) | BAIT | H. sapiens | High | - |
| [NAA40](https://thebiogrid.org/122921/table/homo-sapiens/naa40.html) | BAIT | H. sapiens | High | - |
| [NAF1](https://thebiogrid.org/124938/table/homo-sapiens/naf1.html) | BAIT | H. sapiens | High | - |
| [NBR1](https://thebiogrid.org/110253/table/homo-sapiens/nbr1.html) | BAIT | H. sapiens | High | - |
| [NEU2](https://thebiogrid.org/110832/table/homo-sapiens/neu2.html) | BAIT | H. sapiens | High | 0.957 |
| [NFE2L1](https://thebiogrid.org/110851/table/homo-sapiens/nfe2l1.html) | HIT | H. sapiens | High | - |
| [NFE2L2](https://thebiogrid.org/110852/table/homo-sapiens/nfe2l2.html) | HIT | H. sapiens | High | - |
| [NHLRC2](https://thebiogrid.org/131892/table/homo-sapiens/nhlrc2.html) | BAIT | H. sapiens | High | - |
| [NPM1](https://thebiogrid.org/110929/table/homo-sapiens/npm1.html) | BAIT | H. sapiens | High | - |
| [NR2C2](https://thebiogrid.org/113034/table/homo-sapiens/nr2c2.html) | BAIT | H. sapiens | High | - |
| [NR4A1](https://thebiogrid.org/109407/table/homo-sapiens/nr4a1.html) | BAIT | H. sapiens | High | - |
| [NUDT4](https://thebiogrid.org/116334/table/homo-sapiens/nudt4.html) | HIT | H. sapiens | High | - |
| [NUMA1](https://thebiogrid.org/110980/table/homo-sapiens/numa1.html) | HIT | H. sapiens | High | - |
| [NUP50](https://thebiogrid.org/115982/table/homo-sapiens/nup50.html) | HIT | H. sapiens | High | - |
| [OPTN](https://thebiogrid.org/115436/table/homo-sapiens/optn.html) | BAIT | H. sapiens | High | - |
| [OSBPL10](https://thebiogrid.org/125385/table/homo-sapiens/osbpl10.html) | BAIT | H. sapiens | High | 4.25 |
| [OTUD1](https://thebiogrid.org/128638/table/homo-sapiens/otud1.html) | BAIT | H. sapiens | High | - |
| [OXR1](https://thebiogrid.org/120391/table/homo-sapiens/oxr1.html) | HIT | H. sapiens | High | - |
| [PAF1](https://thebiogrid.org/120081/table/homo-sapiens/paf1.html) | BAIT | H. sapiens | High | 0.5694 |
| [PALB2](https://thebiogrid.org/122843/table/homo-sapiens/palb2.html) | BAIT | H. sapiens | High | 0.9149 |
| [PALM](https://thebiogrid.org/111100/table/homo-sapiens/palm.html) | HIT | H. sapiens | High | - |
| [PDCD6IP](https://thebiogrid.org/115332/table/homo-sapiens/pdcd6ip.html) | HIT | H. sapiens | High | - |
| [PFAS](https://thebiogrid.org/111221/table/homo-sapiens/pfas.html) | HIT | H. sapiens | High | 1.92 |
| [PGAM5](https://thebiogrid.org/128154/table/homo-sapiens/pgam5.html) | HIT | H. sapiens | High | 1.27 |
| [PHF21A](https://thebiogrid.org/119468/table/homo-sapiens/phf21a.html) | BAIT | H. sapiens | High | - |
| [PIDD1](https://thebiogrid.org/120645/table/homo-sapiens/pidd1.html) | HIT | H. sapiens | High | - |
| [PIK3CA](https://thebiogrid.org/111308/table/homo-sapiens/pik3ca.html) | HIT | H. sapiens | High | - |
| [PIN1](https://thebiogrid.org/111317/table/homo-sapiens/pin1.html) | HIT | H. sapiens | High | - |
| [PLEC](https://thebiogrid.org/111355/table/homo-sapiens/plec.html) | HIT | H. sapiens | High | - |
| [POLR2F](https://thebiogrid.org/111431/table/homo-sapiens/polr2f.html) | BAIT | H. sapiens | High | - |
| [POLR3E](https://thebiogrid.org/120840/table/homo-sapiens/polr3e.html) | BAIT | H. sapiens | High | - |
| [PRKAG1](https://thebiogrid.org/111558/table/homo-sapiens/prkag1.html) | BAIT | H. sapiens | High | - |
| [PRKCE](https://thebiogrid.org/111567/table/homo-sapiens/prkce.html) | BAIT | H. sapiens | High | - |
| [PRKCI](https://thebiogrid.org/111570/table/homo-sapiens/prkci.html) | BAIT | H. sapiens | High | - |
| [PRKCZ](https://thebiogrid.org/111576/table/homo-sapiens/prkcz.html) | BAIT | H. sapiens | High | - |
| [PRMT1](https://thebiogrid.org/109512/table/homo-sapiens/prmt1.html) | BAIT | H. sapiens | High | - |
| [PTMA](https://thebiogrid.org/111724/table/homo-sapiens/ptma.html) | BAIT | H. sapiens | High | - |
| [PXN](https://thebiogrid.org/111787/table/homo-sapiens/pxn.html) | HIT | H. sapiens | High | - |
| [PYHIN1](https://thebiogrid.org/127225/table/homo-sapiens/pyhin1.html) | BAIT | H. sapiens | High | - |
| [RAF1](https://thebiogrid.org/111831/table/homo-sapiens/raf1.html) | BAIT | H. sapiens | High | - |
| [RBBP7](https://thebiogrid.org/111866/table/homo-sapiens/rbbp7.html) | BAIT | H. sapiens | High | - |
| [RBM45](https://thebiogrid.org/126210/table/homo-sapiens/rbm45.html) | HIT | H. sapiens | High | 4.71 |
| [RBM5](https://thebiogrid.org/115480/table/homo-sapiens/rbm5.html) | HIT | H. sapiens | High | 0.6757 |
| [RCOR1](https://thebiogrid.org/116796/table/homo-sapiens/rcor1.html) | BAIT | H. sapiens | High | - |
| [RECK](https://thebiogrid.org/114014/table/homo-sapiens/reck.html) | BAIT | H. sapiens | High | - |
| [RETN](https://thebiogrid.org/121192/table/homo-sapiens/retn.html) | BAIT | H. sapiens | High | 0.6511 |
| [RFWD2](https://thebiogrid.org/122136/table/homo-sapiens/rfwd2.html) | BAIT | H. sapiens | High | - |
| [RRAS2](https://thebiogrid.org/116480/table/homo-sapiens/rras2.html) | HIT | H. sapiens | High | - |
| [SASS6](https://thebiogrid.org/127880/table/homo-sapiens/sass6.html) | HIT | H. sapiens | High | 11.54 |
| [SGCE](https://thebiogrid.org/114424/table/homo-sapiens/sgce.html) | HIT | H. sapiens | High | - |
| [SGTA](https://thebiogrid.org/112347/table/homo-sapiens/sgta.html) | HIT | H. sapiens | High | - |
| [SHBG](https://thebiogrid.org/112359/table/homo-sapiens/shbg.html) | HIT | H. sapiens | High | - |
| [SLC38A10](https://thebiogrid.org/125874/table/homo-sapiens/slc38a10.html) | HIT | H. sapiens | High | - |
| [SLK](https://thebiogrid.org/115096/table/homo-sapiens/slk.html) | HIT | H. sapiens | High | 21.2 |
| [SLX4](https://thebiogrid.org/124097/table/homo-sapiens/slx4.html) | BAIT | H. sapiens | High | - |
| [SMARCD1](https://thebiogrid.org/112486/table/homo-sapiens/smarcd1.html) | BAIT | H. sapiens | High | - |
| [SMCHD1](https://thebiogrid.org/116929/table/homo-sapiens/smchd1.html) | HIT | H. sapiens | High | - |
| [SNRNP40](https://thebiogrid.org/114805/table/homo-sapiens/snrnp40.html) | BAIT | H. sapiens | High | - |
| [SNRPN](https://thebiogrid.org/112522/table/homo-sapiens/snrpn.html) | HIT | H. sapiens | High | - |
| [SQSTM1](https://thebiogrid.org/114397/table/homo-sapiens/sqstm1.html) | BAIT | H. sapiens | High | 6.96 |
| [SSH3](https://thebiogrid.org/120299/table/homo-sapiens/ssh3.html) | BAIT | H. sapiens | High | - |
| [STAC2](https://thebiogrid.org/131192/table/homo-sapiens/stac2.html) | HIT | H. sapiens | High | - |
| [STAP2](https://thebiogrid.org/120759/table/homo-sapiens/stap2.html) | HIT | H. sapiens | High | - |
| [STAU1](https://thebiogrid.org/112657/table/homo-sapiens/stau1.html) | BAIT | H. sapiens | High | - |
| [SUGP1](https://thebiogrid.org/121767/table/homo-sapiens/sugp1.html) | HIT | H. sapiens | High | 0.8095 |
| [SYNE4](https://thebiogrid.org/127856/table/homo-sapiens/syne4.html) | BAIT | H. sapiens | High | 0.8932 |
| [TAF1](https://thebiogrid.org/112735/table/homo-sapiens/taf1.html) | BAIT | H. sapiens | High | - |
| [TAX1BP1](https://thebiogrid.org/114405/table/homo-sapiens/tax1bp1.html) | BAIT | H. sapiens | High | - |
| [TBC1D7](https://thebiogrid.org/119412/table/homo-sapiens/tbc1d7.html) | HIT | H. sapiens | High | - |
| [TBKBP1](https://thebiogrid.org/115103/table/homo-sapiens/tbkbp1.html) | HIT | H. sapiens | High | - |
| [TERF1](https://thebiogrid.org/112872/table/homo-sapiens/terf1.html) | BAIT | H. sapiens | High | - |
| [TERF2](https://thebiogrid.org/112873/table/homo-sapiens/terf2.html) | HIT | H. sapiens | High | 0.704 |
| [TET2](https://thebiogrid.org/120151/table/homo-sapiens/tet2.html) | BAIT | H. sapiens | High | - |
| [TLE2](https://thebiogrid.org/112944/table/homo-sapiens/tle2.html) | BAIT | H. sapiens | High | - |
| [TMCO6](https://thebiogrid.org/120647/table/homo-sapiens/tmco6.html) | HIT | H. sapiens | High | - |
| [TMEM160](https://thebiogrid.org/120296/table/homo-sapiens/tmem160.html) | HIT | H. sapiens | High | 4.08 |
| [TNFRSF6B](https://thebiogrid.org/114301/table/homo-sapiens/tnfrsf6b.html) | BAIT | H. sapiens | High | 0.9938 |
| [TOLLIP](https://thebiogrid.org/119978/table/homo-sapiens/tollip.html) | BAIT | H. sapiens | High | - |
| [TRIM15](https://thebiogrid.org/124626/table/homo-sapiens/trim15.html) | BAIT | H. sapiens | High | - |
| [TRIM28](https://thebiogrid.org/115457/table/homo-sapiens/trim28.html) | BAIT | H. sapiens | High | - |
| [TRIM37](https://thebiogrid.org/110678/table/homo-sapiens/trim37.html) | BAIT | H. sapiens | High | - |
| [TRIM67](https://thebiogrid.org/136847/table/homo-sapiens/trim67.html) | BAIT | H. sapiens | High | - |
| [TRMT61B](https://thebiogrid.org/120338/table/homo-sapiens/trmt61b.html) | HIT | H. sapiens | High | 0.9954 |
| [TSC22D4](https://thebiogrid.org/123563/table/homo-sapiens/tsc22d4.html) | HIT | H. sapiens | High | 7.07 |
| [TSPAN5](https://thebiogrid.org/115405/table/homo-sapiens/tspan5.html) | BAIT | H. sapiens | High | 0.965 |
| [TUBG1](https://thebiogrid.org/113134/table/homo-sapiens/tubg1.html) | BAIT | H. sapiens | High | - |
| [TUSC2](https://thebiogrid.org/116462/table/homo-sapiens/tusc2.html) | HIT | H. sapiens | High | - |
| [UBC](https://thebiogrid.org/113164/table/homo-sapiens/ubc.html) | BAIT | H. sapiens | High | - |
| [UBE2A](https://thebiogrid.org/113167/table/homo-sapiens/ube2a.html) | BAIT | H. sapiens | High | - |
| [UIMC1](https://thebiogrid.org/119697/table/homo-sapiens/uimc1.html) | HIT | H. sapiens | High | - |
| [USH1G](https://thebiogrid.org/125876/table/homo-sapiens/ush1g.html) | HIT | H. sapiens | High | - |
| [USP11](https://thebiogrid.org/113866/table/homo-sapiens/usp11.html) | BAIT | H. sapiens | High | 1.63 |
| [VGLL3](https://thebiogrid.org/132991/table/homo-sapiens/vgll3.html) | HIT | H. sapiens | High | - |
| [VWA8](https://thebiogrid.org/116710/table/homo-sapiens/vwa8.html) | HIT | H. sapiens | High | 20.8 |
| [WDR82](https://thebiogrid.org/123245/table/homo-sapiens/wdr82.html) | BAIT | H. sapiens | High | - |
| [WDR83](https://thebiogrid.org/124019/table/homo-sapiens/wdr83.html) | HIT | H. sapiens | High | - |
| [WDTC1](https://thebiogrid.org/116677/table/homo-sapiens/wdtc1.html) | BAIT | H. sapiens | High | - |
| [WDYHV1](https://thebiogrid.org/120405/table/homo-sapiens/wdyhv1.html) | HIT | H. sapiens | High | - |
| [WWTR1](https://thebiogrid.org/117434/table/homo-sapiens/wwtr1.html) | BAIT | H. sapiens | High | - |
| [XPO1](https://thebiogrid.org/113348/table/homo-sapiens/xpo1.html) | BAIT | H. sapiens | High | - |
| [ZC3H18](https://thebiogrid.org/125858/table/homo-sapiens/zc3h18.html) | BAIT | H. sapiens | High | 0.9156 |
| [ZNF121](https://thebiogrid.org/113474/table/homo-sapiens/znf121.html) | HIT | H. sapiens | High | - |
| [ZNF213](https://thebiogrid.org/113543/table/homo-sapiens/znf213.html) | BAIT | H. sapiens | High | 0.956 |
| [ZRANB1](https://thebiogrid.org/120139/table/homo-sapiens/zranb1.html) | BAIT | H. sapiens | High | 3.76 |
| [ZSCAN32](https://thebiogrid.org/120265/table/homo-sapiens/zscan32.html) | HIT | H. sapiens | High | - |

**Supplementary Table 9. Clinical characteristics of 180 GC patients who underwent surgical treatment in the presence of TMEM160 expression**

| **Variables** | **N** | **TMEM160** | | |
| --- | --- | --- | --- | --- |
|  |  | Low(%) | High(%) | P value |
| **Gender** |  |  |  | 0.341 |
| **Male** | 94 | 46(48.9%) | 48(51.1%) |  |
| **Female** | 86 | 36(41.9%) | 50(58.1%) |  |
| **Age(years)** |  |  |  | 0.603 |
| ≥ 60 | 96 | 42(43.8%) | 54(56.3%) |  |
| ＜60 | 84 | 40(47.6%) | 44(52.4%) |  |
| **Tumor size (cm)** |  |  |  | 0.265 |
| ≥4 | 85 | 35(41.2%) | 50(58.8%) |  |
| <4 | 95 | 47(49.5%) | 48(50.5%) |  |
| **Lauren type** |  |  |  | 0.357 |
| Intestinal type | 83 | 39(47.0%) | 44(53.0%) |  |
| Diffuse type | 97 | 43(44.3%) | 54(55.7%) |  |
| **Differentiation status** |  |  |  | 0.473 |
| Poor and undifferentiated | 109 | 52(47.7%) | 57(52.3%) |  |
| Well + Moderate | 71 | 30(42.3%) | 41(57.7%) |  |
| **Depth of invasion** |  |  |  | 0.002 |
| T3 + T4 | 120 | 45(37.5%) | 75(62.5%) |  |
| T1 + T2 | 60 | 37(61.7%) | 23(38.3%) |  |
| **Lymph node metastasis** |  |  |  | 0.009 |
| N1 + N2 + N3 | 139 | 56(40.3%) | 83(59.7%) |  |
| N0 | 41 | 26(63.4%) | 15(36.6%) |  |
| **TNM stage** |  |  |  | <0.001 |
| III | 120 | 42(35.0%) | 78(65.0%) |  |
| I + II | 60 | 40(66.7%) | 20(33.3%) |  |

**Supplementary Table 10. Univariate and Multivariate COX regression analysis of prognostic factors associated with OS based on 180 GC patients who underwent surgical treatment**

| **Variables** | **Univariate analysis** | | **Multivariate analysis** | |
| --- | --- | --- | --- | --- |
|  | **HR (95% CI)** | **P value** | **HR (95% CI)** | **P value** |
| **Gender (female vs. male)** | 1.456(0.983-2.157) | 0.061 | – | – |
| **Age (years) (≥ 60 vs. < 60)** | 1.189(0.803-1.760) | 0.388 | – | – |
| **Tumor size (cm) (< 4 vs. ≥ 4)** | 0.906(0.611-1.341) | 0.621 | – | – |
| **Differentiation status (well + moderate vs. poor and undifferentiated)** | 0.856(0.570-1.285) | 0.453 | – | – |
| **Lauren type (diffuse type vs. intestinal type)** | 0.887(0.599-1.312) | 0.548 |  | – |
| **Depth of invasion (T1 + T2 vs. T3 + T4)** | 0.207(0.115-0.373) | <0.001 | 0.390(0.210-0.724) | 0.037 |
| **Lymph node metastasis (N0 vs. N1 + N2 + N3)** | 0.243(0.122-0.482) | <0.001 | 1.620(0.436-2.014) | 0.347 |
| **TNM stage (I + II vs. III)** | 0.181(0.099-0.332) | <0.001 | 0.199(0.062-0.638) | <0.001 |
| **TMEM160(high vs. low)** | 2.864(1.880-4.361) | <0.001 | 2.101(1.370-3.221) | 0.001 |
| **NRF2 (high vs. low)** | 2.028(1.339-3.071) | 0.001 | 1.981(1.293-3.035) | 0.002 |

| **Variables** | **N** | **TMEM160** | | |
| --- | --- | --- | --- | --- |
|  |  | Low(%) | High(%) | P value |
| **Gender** |  |  |  | 0.887 |
| Male | 60 | 32(53.3%) | 28(46.7%) |  |
| Female | 29 | 15(51.7%) | 14(48.3%) |  |
| **Age(years)** |  |  |  | 0.072 |
| ≥ 60 | 45 | 28(62.2%) | 17(37.8%) |  |
| <60 | 44 | 19(43.2%) | 25(56.8%) |  |

**Supplementary Table 11. Clinical characteristics of 89 advanced GC patients in the presence of TMEM160 expression**

**Supplementary Table 12. Univariate and Multivariate COX regression analysis of prognostic factors associated with OS based on 89 advanced GC patients**

| **Variables** | **Univariate analysis** | | **Multivariate analysis** | |
| --- | --- | --- | --- | --- |
|  | **HR (95% CI)** | **P value** | **HR (95% CI)** | **P value** |
| **Gender (female vs. male)** | 1.241(0.786-1.960) | 0.354 | – | – |
| **Age (years) (≥ 60 vs. < 60)** | 0.540(0.346-0.845) | 0.007 | 1.126(0.731-1.434) | 0.144 |
| **TMEM160(high vs. low)** | 5.424(3.259-9.028) | <0.001 | 3.550(1.926-6.441) | <0.001 |
| **NRF2 (high vs. low)** | 4.391(2.665-7.233) | <0.001 | 2.587(1.424-4.701) | 0.002 |

**Supplementary Table 13. Abbreviations in this research**

| GC | Gastric cancer |
| --- | --- |
| TMEM160 | Transmembrane protein 160 |
| GPX4 | Glutathione peroxidase 4 |
| KEAP1 | Kelch-like ECH-associated protein 1 |
| NRF2 | Nuclear factor erythroid 2-related factor 2 |
| SLC7A11 | Solute carrier family 7a member 11 |
| TRIM37 | Tripartite motif-containing protein |
| FBS | Fetal bovine serum |
| RT-qPCR | Real-time quantitative polymerase chain reaction |
| WB | Western blotting |
| MDA | Malondialdehyde |
| TEM | Transmission electron microscopy |
| ROS | Reactive oxygen species |
| CCK-8 | Cell Counting Kit-8 |
| PFA | Paraformaldehyde |
| IHC | Immunohistochemistry |
| IF | Immunofluorescence |
| CDX | Cell-line-derived xenograft |
| PDX | Patient-derived xenograft |
| PBS | Phosphate buffer solution |
| Co-IP | Co-Immunoprecipitation |
| CQ | Chloroquine |
| Cul3 | Cullin 3 |
| BAP1 | Breast cancer susceptibility gene 1-associated protein 1 |
| AMER1 | APC membrane recruitment protein1 |
| ARF | Alternative Reading Frame |
| MLL4 | Mixed linked leukemia 4 |
| IC_50_ | Half maximal inhibitory concentration |
| PD-L1 | Programmed cell death 1 ligand 1 |
| SPOP | Speckle-type POZ (pox virus and zinc finger protein) protein |
| OS | Overall Survival |
| OD | Optical density |
| HR | Hazard ratio |
| PD | Disease progression |
| PR | Partial response |
| 95% CI | 95% confidence interval |
| 5-fu | 5-Fluorouraci |
