# Supplementary material for: TMEM160 inhibits KEAP1 to suppress ferroptosis and induce chemoresistance in gastric cancer
Source: Cell Death Dis. 2025 Apr 13;16(1):287. doi: 10.1038/s41419-025-07621-0 (PMC11994801; doi:10.1038/s41419-025-07621-0)
Supplement: Supplementary file 4 — Raw blots (the raw blots image) [file 41419_2025_7621_MOESM4_ESM.docx]

**The raw blots for this study are as follows:**

**Fig. 1B Fig.1C**


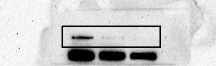

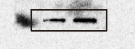


TMEM160

TMEM160


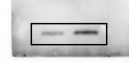

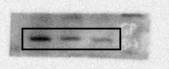


GPX4

GPX4


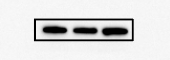

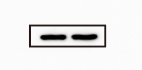


GAPDH

GAPDH

**Fig.3F Fig.3G**


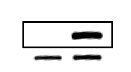
**
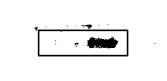
**

IB:HA

IB:HA


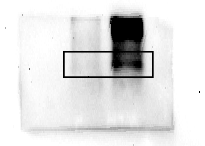

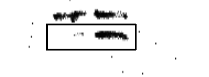


IB:Myc

IB:Myc


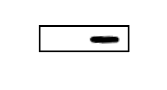

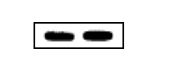


HA

HA


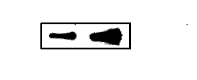

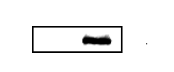


Myc

Myc

**Fig.3H Fig.3I**

**
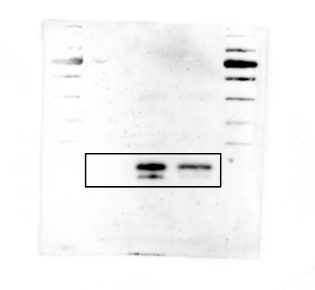
**


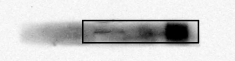

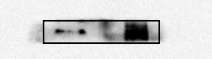
 HGC-27 BGC-823

TMEM160

His


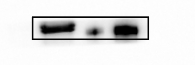

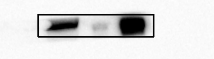


KEAP1


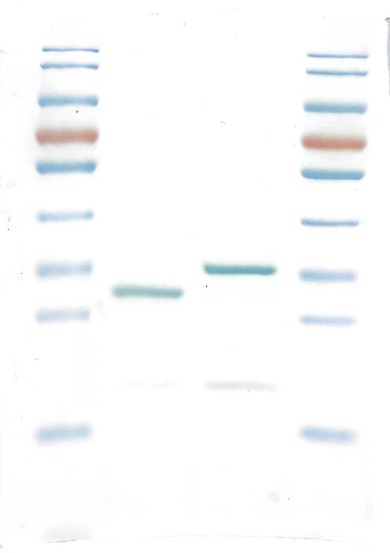


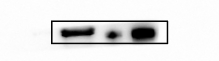

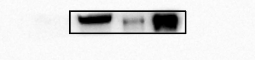


KEAP1


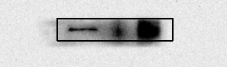

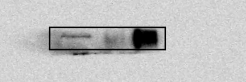


TMEM160

**Fig.3K**


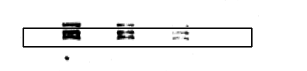
 HEK-293T

IB:Myc


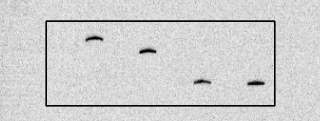
IB:Flag


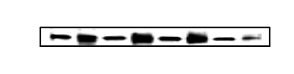
Myc


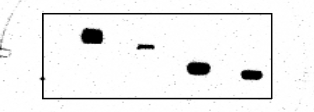
Flag

**
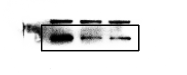

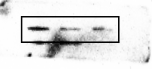
Fig.4A Fig.4B**

**
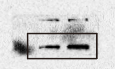
**TMEM160

TMEM160


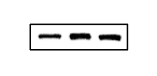


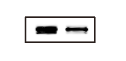

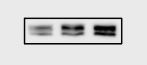


KEAP1


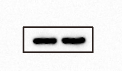

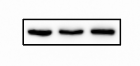
KEAP1


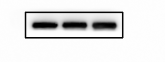


GAPDH

GAPDH

BGC-823 HGC-27 SNU-216

**Fig.4F**


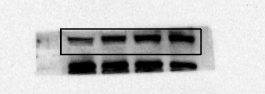


TMEM160


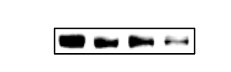


KEAP1


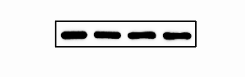
GAPDH

**Fig.4G Fig.4H**

BGC-823 SNU-216


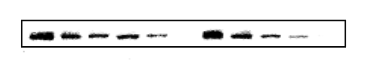

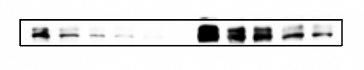


KEAP1

KEAP1


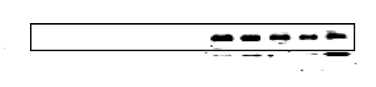

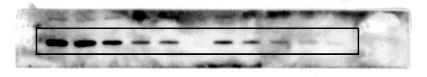


Myc

TMEM160


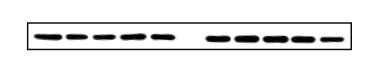

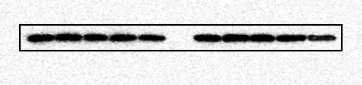


GAPDH

GAPDH

**
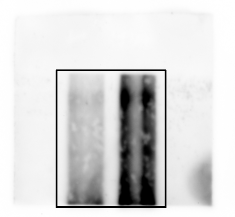
Fig.4I**

IB:Ub


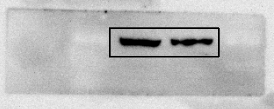


HA


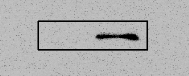


Myc


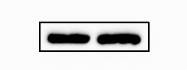


GAPDH

**
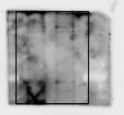

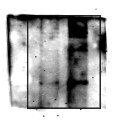

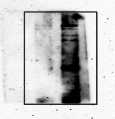

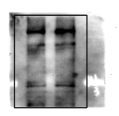

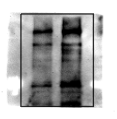

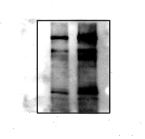
Fig.4J Fig.4K**

IB:Ub

IB:Ub


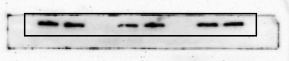


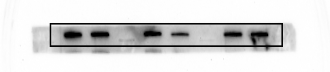


IB:HA

IB:HA


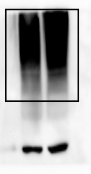

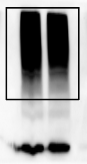

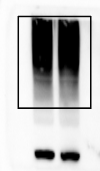

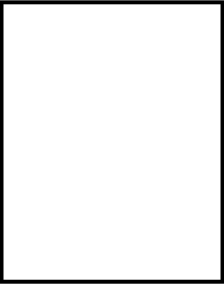

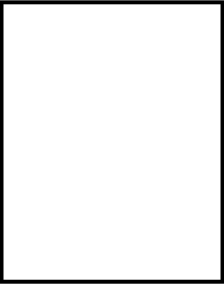

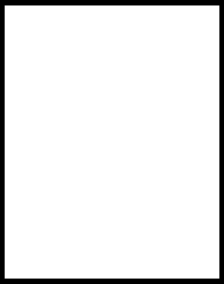

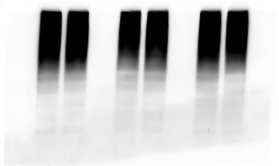


Ub

Ub


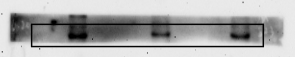

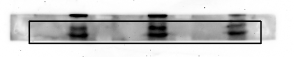


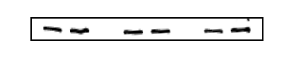
Myc

Myc


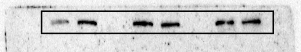


HA

HA


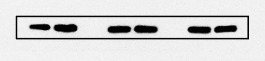


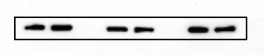
GAPDH

GAPDH

**Fig.5A Fig.5B**

**
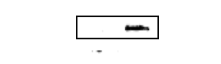
**
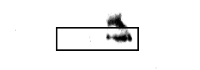


IB:Myc

IB:Myc


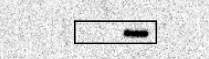

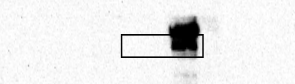


IB:Flag

IB:Flag


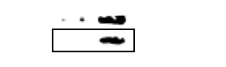

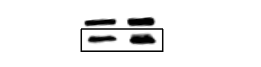


Myc

Myc


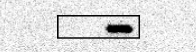

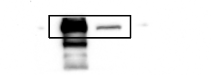


Flag

Flag

**
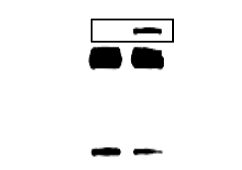
Fig.5C**

**Fig.5D**

**
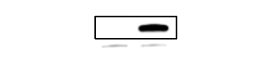
**IB:HA

IB:HA


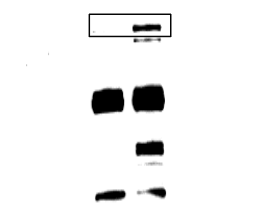

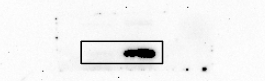
IB:Flag

IB:Flag


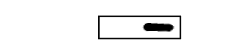


HA


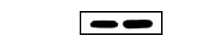


HA


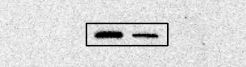

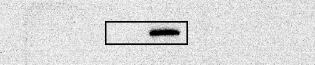


Flag

Flag

**Fig.5E**


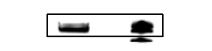
 BGC-823 BGC-823


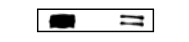
KEAP1

KEAP1


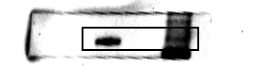

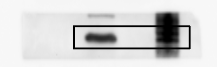


TMEM160

TMEM160


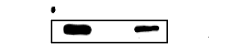

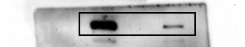


TRIM37

TRIM37

**Fig.5F**

HGC-27

HGC-27


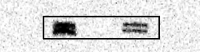

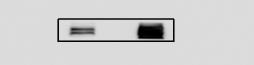


KEAP1

KEAP1


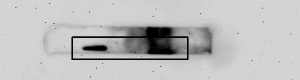

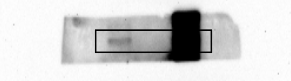


TMEM160

TMEM160


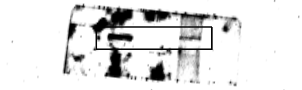

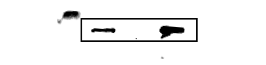
TRIM37

TRIM37

**Fig.5G Fig.5H**


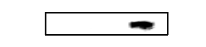


IB:Myc

Flag

IB:Flag

IB:HA

KEAP1

Myc

GAPDH

Flag

HA

GAPDH

**Fig.5I Fig.5K**

KEAP1

IB:UB

Flag

GAPDH

Myc

**Fig.5J**

Flag

KEAP1

HA

TRIM37

GAPDH

GAPDH

**Fig.5L Fig.5M**

IB:Ub

IB:Ub

IB:HA

IB:HA

Ub

Ub

Flag

Flag

HA

HA

GAPDH

GAPDH

**Fig.6B Fig.6C**

TMEM160

TMEM160

NRF2

NRF2

SLC7A11

SLC7A11

GPX4

GPX4

GAPDH

GAPDH

BGC-823 HGC-27 SNU-216

**Fig.6G**

BGC-823 HGC-27

Myc

Myc

NRF2

NRF2

SLC7A11

SLC7A11

GPX4

GPX4

GAPDH

GAPDH

**Fig.7E Fig.7J**

TMEM160

TMEM160

KEAP1

KEAP1

NRF2

NRF2

SLC7A11

SLC7A11

GPX4

GPX4

GAPDH

GAPDH

**Supplementary Fig.2A Supplementary Fig.2B**

TMEM160

TMEM160

GAPDH

GAPDH

**Supplementary Fig.2C**

TMEM160

GAPDH

**Supplementary Fig.5A Supplementary Fig.5B**

TMEM160

TMEM160

GAPDH

GAPDH
